# Straw retention drives microbial community succession to improve soil C/N cycling: insights from a multi-year rice-based system

**DOI:** 10.3389/fmicb.2025.1590788

**Published:** 2025-05-20

**Authors:** Shu Jia, Yue-Dong Li, Hang Qu, Bo Li, Ying-hua Juan, Yue-hua Xing, Yan Liu, Hong-jing Bao, Wen-tao Sun

**Affiliations:** ^1^Institute of Plant Nutrition and Environmental Resources, Liaoning Academy of Agricultural Sciences, Shenyang, China; ^2^Liaoning Rice Research Institute, Liaoning Academy of Agricultural Sciences, Shenyang, China

**Keywords:** rice straw retention, soil microbial community, microbial function, carbon cycle, nitrogen cycle

## Abstract

The soil microbial community plays a crucial role in driving the decomposition and mineralization of plant residues, thereby affecting carbon (C) and nitrogen (N) cycling and storage. Straw retention provides soil with C and N sources, which enhances microbial community composition and nutrient cycling. While long-term straw retention has been shown to improve soil quality and nutrient-use efficiency, the impacts of short-term straw-return treatment on soil quality and the underlying microbiological mechanism of straw in improving soil fertility and nutrient-use efficiency remain unclear. The present study aimed to elucidate the dynamic responses of soil microbial community structure and function to rice straw retention using a multi-year field experiment. The findings revealed that rice straw returned for 3 and 5 consecutive years (S3 and S5, respectively), enhanced soil organic carbon (SOC) and available phosphorous (AP) contents, increased fungal biomass, and stimulated the growth of cellulose-decomposing microbial communities. Furthermore, S3 and S5 treatments increased the activities of C cycling enzymes (β-xylosidase) and N cycling enzymes (N-acetyl-glucosaminidase and urease). These treatments also increased the genes abundance associated with C-cycling (*sdimo*), nitrification (*amoA* and *amoB*), and N fixation (*nifH*), while enriched genes related to C cycling and N metabolism pathways (nitrification and nitrate reduction). In contrast, the abundance of genes involved in denitrification (*nirS*) was reduced. However, S3 and S5 treatments led to an increased abundance of the plant pathogens *Magnaporthe oryzae* and *Ustilaginoidea virens*. This work demonstrates that short-term straw retention effectively enhances soil microecological environment and microbial functionality and also underscores the need for strategies to mitigate pathogen accumulation for sustainable agricultural practices.

## Graphical abstract

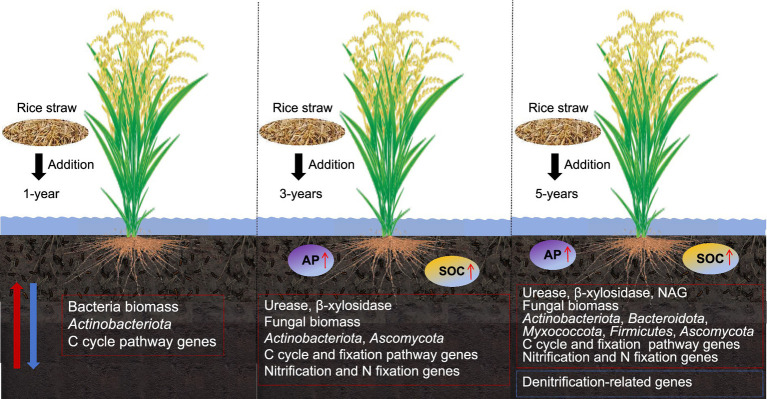

## 1 Introduction

Soil microorganisms are vital biological indicators for assessing agricultural soil quality and ecosystem functionality (Coban et al., 2022; Yuan et al., 2024; Pang et al., 2024). They play a crucial role in sustainable crop productivity by facilitating nutrient cycling and organic matter decomposition, enhancing plant immunity, and contributing to soil remediation (Klimasmith and Kent, 2022). As the most active components of soil microecosystems, microorganisms are highly sensitive to environmental changes and agricultural interventions, such as tillage and fertilization (Liu et al., 2020; Yin et al., 2025; Zhang et al., 2023). Different fertilizer application patterns and management practices directly impact crop yields and significantly drive changes in the composition and function of soil microbial communities (Tang et al., 2023; Yang et al., 2025).

Rice is one of the most important food crops globally and serves as the main source of food for more than half of global population. The stable production of rice is crucial for global food security. With the continuous growth of the world economy and population, the demand for both rice yield and quality has steadily increased (Yang et al., 2019). To enhance rice production, large quantities of fertilizers are widely utilized. However, the prolonged use of chemical fertilizers in agriculture adversely impacts soil quality and microecology (Huang Y. P. et al., 2021; Yang et al., 2022), ultimately affecting soil productivity and health (Wang et al., 2022).

Several studies have shown that the prolonged use of chemical fertilizers, particularly nitrogen (N), significantly reduces soil microbial diversity and biomass, disrupts the stability of the bacterial community structures (Wang et al., 2018), and adversely affects soil microbial N fixation and signal transduction functions (Li et al., 2020). Furthermore, excessive fertilizer application can enrich ammonia oxidation functional genes in soil microorganisms, leading to nitrate accumulation, leaching, and increased N2O emissions, ultimately affecting soil nutrient-use efficiency and farmland productivity (Bai et al., 2022; Lin et al., 2025). Therefore, achieving sustainable agricultural production requires the implementation of scientific fertilization techniques that can improve soil quality and soil microecological environment while ensuring rice yield.

The long-term application of organic fertilizers, including animal manure, green manure, and crop straw, can significantly increase soil nutrient content (Cui et al., 2024) and microbial biomass (Cui et al., 2023), improve soil microbial structures, and promote soil nutrient cycling and utilization (Zhou J. S. et al., 2024; Zhou G. P. et al., 2024). In China, crop straw resources are abundant, and straw is rich in essential nutrients, such as N, phosphorus (P), and potassium (K). When returned to the field, straw can mitigate the need for extensive chemical fertilizer application (Liu J. A. et al., 2021). Straw retention facilitates the transport of carbon (C) and nitrogen (N) sources to the soil, thereby improving microbial activity, composition, and function (Yuan et al., 2023). This process increases microbial functional diversity (Xu et al., 2023) and improves soil nutrient availability (Wang Y. J. et al., 2020), making it an effective strategy for sustainable agriculture.

Despite the positive effects of returning straw to the field, existing research has primarily focused on long-term (more than 10 years) straw-return treatment, while studies investigating the impacts of short-term (less than 10 years) straw-return treatments on soil quality and microecology remain limited. Additionally, the microbiological mechanisms through which straw improves soil fertility and nutrient-use efficiency are still poorly understood. The objective of this study was to evaluate the effects of rice straw retention on soil properties, soil microbial activities, and the dynamic responses of soil microbial community structures and potential functions under continuous straw retention using a 1-, 3-, and 5-year rice straw retention field experiment. We aimed to characterize straw-responsive microbial communities that drive soil nutrient cycling and elucidate their functional mechanisms in improving soil fertility and nutrient-use efficiency. Genomic DNA extracted from soil samples was subjected to: (i) quantitative PCR (qPCR) for microbial abundance quantification, (ii) Illumina MiSeq platform-based 16S rRNA gene sequencing for microbial community profiling, and (iii) shotgun metagenomic sequencing for functional gene analysis, enabling comprehensive tracking of microbial succession and characterization of key genes involved in carbon and nitrogen cycling processes. The soil physicochemical properties and enzyme activities related to C and N cycling were also elucidated. Furthermore, we assessed changes in the abundance of potential plant pathogens. This study provides a theoretical foundation for the fertilizer utilization of straw resources and promotes sustainable agricultural development.

## 2 Materials and methods

### 2.1 Experimental design and sample collection

The experimental field was in Tongerpu Town, Dengta City, Liaoyang City, Liaoning Province, China (41° 45′N, 123° 04′E). The region is characterized by a temperate, northern continental climate with an average annual precipitation of 500–700 mm, an average annual temperature of 6–8°C, and a frost-free period of approximately 150 days. The tested field soil comprised Hydragric Anthrosols; its physical and chemical properties are listed in Supplementary Table S1.

The experimental field involved four treatments (each treatment area was 348.84 m^2^, i.e., 22.80 × 15.30 m): (1) control (SCK): rice straw was not returned to the field; (2) S1: rice straw was returned to the field for 1 year (2022); (3) S3: rice straw was returned to the field consecutively for 3 years (2020–2022); and (4) S5: the straw was returned consecutively to the field for 5 years (2018–2022). The experiment was arranged in a randomized complete block design (RCBD) with three replicates, where all treatments were randomly assigned within each block to account for field variability. This study used the rice variety Liaojing 401, which was planted using a rice transplanter. Seedlings were transplanted at a row spacing of 33.33 × 18.00 cm. Each year, rice seedlings were transplanted in early May, and rice plants were harvested in early October. After harvest, the rice straw was chopped into approximately 10 cm pieces and then directly returned to the field for rotational tillage. The amount of returned straw was 9.0 t ha^−1^ with the following fertilizer application rates: N 210 kg ha^−1^, P_2_O_5_ 60 kg ha^−1^, and K_2_O 120 kg ha^−1^. Chemical N fertilizer was applied as a basal, tillering, and panicle fertilizer (application ratio: 5:3:2). Chemical K_2_O and P_2_O_5_ were also applied as basal fertilizers. The chemical N, P, and K fertilizers were urea, potassium dihydrogen phosphate, and potassium chloride. In mid-October 2023, five topsoil cores (5 cm diameter × 20 cm depth; free from visible crop roots) were systematically collected from inter-rows of each plot using a stainless steel corer and homogenized to form one composite sample per plot. A total of 12 fresh soil samples were collected (four treatments × three replicates), transported on ice to the laboratory within 1 h, and immediately passed through a 2-mm mesh to remove roots and rocks. The soil was then divided into three parts: (1) immediately stored at −80°C for molecular analyses (high-throughput sequencing analysis, metagenomic sequencing, and qRT-PCR analysis), (2) preserved at 4°C for enzyme activity assays within 2 h; and (3) air-dried (25°C, 7 d) for physicochemical characterization.

For enzyme activity measurements, we quantified the activities of carbon-cycling enzymes (β-glucosidase [BG], cellobiohydrolase [CBH], and β-xylosidase) and nitrogen-cycling enzymes (N-acetyl-glucosaminidase [NAG], L-leucine aminopeptidase [LAP], and urease) using commercial kits (Shanghai Enzyme-linked Biotechnology, China) following the manufacturer’s protocol with the following modifications: soil suspensions (1:10 w/v in 50 mM acetate buffer, pH 5.5) were incubated at 37°C for 2–4 h depending on the enzyme, with reactions terminated by alkaline solution (0.5 M NaOH) before spectrophotometric analysis.

### 2.2 DNA extraction and qRT-PCR

Soil DNA was extracted from fresh soil (0.5 g) using an E.Z.N.A.^®^ soil DNA Kit (Omega Bio-tek, Norcross, GA, United States) according to the manufacturer’s instructions. The concentration and quality of extracted DNA were evaluated using an ND-2000 spectrophotometer (Thermo Fisher Scientific, Wilmington, DE, United States).

The gene absolute abundance of the bacteria, fungi, and other functional microorganisms related to C and N cycling were evaluated using qRT-PCR. The selected genes for this evaluation included 16S rRNA and ITS rRNA, as well as genes associated with C cycling, specifically *cbhI* (encoding cellobiohydrolase I, EC 3.2.1.91, catalyzing cellulose hydrolysis), *GH48* (glycoside hydrolase family 48 gene, involved in crystalline cellulose degradation), and *sdimo* (soluble diiron monooxygenase gene, mediating aromatic compound oxidation). For N cycling, the examined genes included *nifH* (nitrogenase reductase, EC 1.18.6.1, for biological N₂ fixation), *amoA* and *amoB* (ammonia monooxygenase subunits, EC 1.7.3.4, for nitrification), *narG* (respiratory nitrate reductase, EC 1.7.99.4, for nitrate reduction), *nirK* and *nirS* (copper- and cytochrome cd₁-type nitrite reductases, EC 1.7.2.1, for denitrification), and *nosZ* (nitrous oxide reductase, EC 1.7.2.4, for N₂O reduction). To evaluate the effects of continuous straw return on the abundance of rice pathogenic fungi in soil, we quantified the gene copy number of *Magnaporthe oryza* and *Ustilaginoidea virens*. Gene copy numbers (absolute abundance) were determined using qRT-PCR with a Line-Gene 9600 Plus real-time PCR detection system (Bioer, Hangzhou, China). Detailed information regarding the primers and qRT-PCR conditions is provided in the Supplementary Table S2 (Li et al., 2011, Sun et al., 2013, Yang et al., 2021). Each sample was amplified in triplicate. Gene copy numbers were calculated using standard curves generated by amplifying known DNA quantities from recombinant plasmids carrying each target.

### 2.3 High-throughput sequencing analysis

To explore the diversity and structures of the soil bacteria and fungi, DNA from the soil samples was sequenced using an Illumina MiSeq 300 platform (Illumina, San Diego, United States). The primer pairs 338F/806R and ITS1/ITS2 were used for bacteria and fungi, respectively (Supplementary Table S2). Sequence reads were demultiplexed, quality-filtered using fastp (version 0.20.0; Chen et al., 2018), and merged using FLASH (version 1.2.11^1^). The operational taxonomic units (OTUs) were clustered with a 97% similarity cutoff using UPARSE (version 7.1^2^). Chimeric sequences were identified and removed using UCHIME (Edgar 2013). The taxonomy of each gene sequence was analyzed using the RDP Classifier (version 2.11^3^) against the Silva (Release138) and Unite (Release 8.0^4^) databases, with a 70% ^5^confidence threshold.

### 2.4 Shotgun metagenomic sequencing

DNA extracts were fragmented to an average of 400 bp using Covaris M220 (Gene Company Limited, China) for paired-end library construction. The paired-end library was constructed using NEXTFLEX^®^ Rapid DNA-Seq (Bioo Scientific, Austin, TX, United States). Adapters containing the full complement of sequencing primer hybridization sites were ligated to the blunt-ends of the fragments. Paired-end sequencing was performed on Illumina NovaSeq (Illumina Inc., San Diego, CA, United States) at Majorbio Bio-Pharm Technology Co., Ltd. (Shanghai, China) using a NovaSeq 6000 S4 Reagent Kit v1.5 (300 cycles) according to the manufacturer’s instructions.^6^

### 2.5 Statistical analyses

The data were analyzed using the freely available Majorbio Cloud Platform.^7^ The Mothur software was used to calculate the alpha diversity index using random sampling. Alpha diversity and richness were determined at the OTU level using the Shannon and Chao1 indices, respectively. Analysis of variance was used to compare the statistical differences among groups. Significant differences were identified at the 95% confidence level. The *p* value was corrected using a multiple test with false discovery rate (FDR) and results with *p* < 0.05 were considered statistically significant. Similarities in the samples in terms of microbial taxa (bacteria and fungi) and profiles of gene family relative abundance (Kyoto Encyclopedia of Genes and Genomes [KEGG] and Carbohydrate-Active Enzyme [CAZy] databases) were measured using the unweighted UniFrac distance for phylogenetic relationships and the Bray–Curtis dissimilarity index for gene families. A one-way analysis of the similarity test with 999 permutations was conducted to evaluate statistical significance. The Mann–Whitney U-test was used to evaluate differences in two-group comparisons. The *p*-value was corrected using multiple tests with a false discovery rate. Redundancy analysis (RDA) and Spearman’s correlation analysis were used to study the correlations between soil environmental factors, microbial community modifications, and microbial functionality induced by straw return over several consecutive years. Unless otherwise stated, statistical analyses were conducted, and plots were generated using R software (version 3.3.1).

## 3 Results

### 3.1 Soil physicochemical properties and enzyme activities under straw retention

Total N (TN), total P (TP), total K (TK), and available N (AN; *p* > 0.05) tended to improve with straw return, whereas soil organic C (SOC) and available phosphorous (AP) contents were significantly increased in S3 and S5 (*p* < 0.05; Supplementary Table S1). Straw retention also had a significant positive effect on the activities of C and N cycle-related enzymes (Supplementary Table S3), and β-xylosidase, NAG, and urease were increased by 69.39, 45.12, and 17.41% compared with the levels in the CK, respectively (*p* < 0.05).

### 3.2 Microbial communities under straw retention

Compared with CK, the gene copy number of the 16S rRNA in S1 increased significantly (*p* < 0.05), while S3 and S5 returned to levels comparable to CK, with no significant differences (*p* > 0.05, Supplementary Table S4). In S3 and S5, the absolute abundance of the ITS rRNA gene was significantly higher (*p* < 0.05). These results revealed that straw retention for 3 and 5 years significantly increased fungal biomass but not bacterial biomass.

After quality filtering, a total of 574,880 bacteria 16Sv3-v4 and 782,583 fungal ITS1 high-quality sequences were obtained for the soil samples from four treatments. Subsequently, 49,124 bacterial and 5,517 fungal operational taxonomic units (OTUs) were assembled at a 97% confidence interval (Supplementary Table S5). For bacterial communities, S1 exhibited significantly reduced richness compared to CK (*p* < 0.05, Supplementary Figure S1), while no significant differences were observed in either richness or diversity among CK, S3, and S5 (*p* > 0.05). For fungal communities, the diversity and evenness in S5 was significantly lower than that in CK (*p* < 0.05), with no significant differences in richness or diversity detected among CK, S1, and S3 (*p* > 0.05).

**Figure 1:**
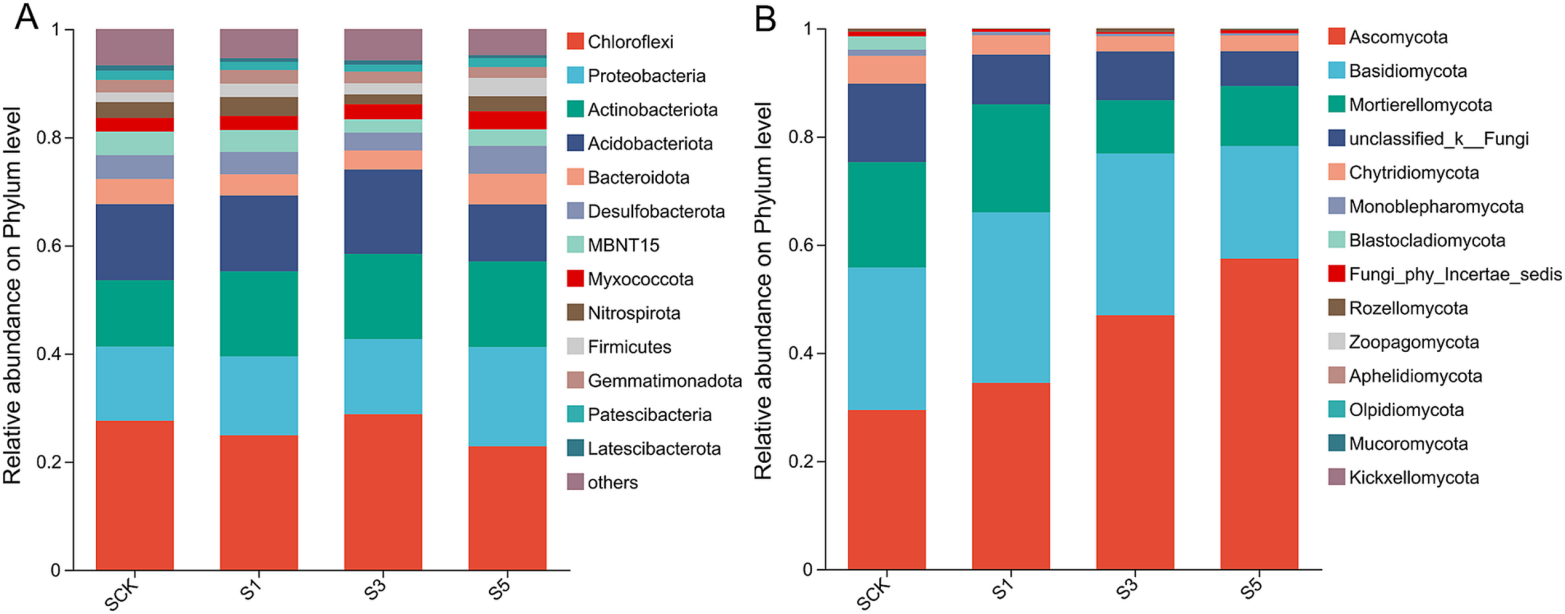
Relative abundances of the bacterial **(A)** and fungal **(B)** phyla in different treatments. SCK, the straw was not returned to the field; S1, S3, and S5, the straw was returned to the field consecutively for 1, 3, and 5 years, respectively.

The predominant bacterial phyla (>1%) in the soil included Chloroflexi, Proteobacteria, Actinobacteriota, Acidobacteriota, Bacteroidota, Desulfobacterota, Myxococcota, Nitrospirota, Firmicutes, Gemmatimonadota, Patescibacteria, Latescibacterota, and “others” (bacteria with an abundance of < 1%; Figure 1A). Straw retention altered the compositions of the microbial communities (Supplementary Table S6); retention for 1 year significantly increased the abundance of Actinobacteria (*p* < 0.05), and retention for 5 years significantly increased the abundances of Proteobacteria, Bacteroidetes, Myxococcus, and Firmicutes (*p* < 0.05) and significantly decreased that of Latescibacterota (*p* < 0.05). At the genus level, straw retention for 5 years significantly increased the abundance of *Trichococcus* and *Bradyrhizobium* (Supplementary Table S7).

The predominant fungal phyla (>1%) in the soil were Ascomycota, Basidiomycota, Mortierellomycota, Chytridiomycota, Monoblepharomycota, Blastocladiomycota, Rozellomycota, and “others” (fungi with an abundance of <1%; Figure 1B). Straw retention for 5 years significantly increased the abundance of Ascomycota and significantly decreased that of Monoblepharomycota (*p* < 0.05; Supplementary Table S6). At the genus level, straw retention for 1 year significantly increased the abundances of *Mortierella*, *Mrakia*, and *Glaciozymade* and significantly decreased the abundance of *Tausonia*. All abundances returned to CK levels after 3 and 5 years (Supplementary Table S7). Straw retention for 3 and 5 years also stimulated the growth of *Pseudeurotium*, *Pseudogymnoascus*, *Sordariales*, *Trichosporiella*, *Achroiostachys*, *Clavidisculum*, and *Schizothecium* but hindered the growth of *Stellatospora* and *Linnemannia* (*p* < 0.05). Furthermore, straw retention for 5 years significantly increased the absolute abundances of *M. oryza* and *U. virens* by 348.65 and 524.73%, respectively, compared with the CK (Supplementary Table S4). Correlation network analysis of fungal communities and pathogenic fungi (Supplementary Figure S3) revealed that the abundance of *U. virens* was significantly positively correlated with *Ustilaginoidea*, *Tausonia*, *Mortierellaceae*, and *Spizellomycetales* (|*r*| > 0.5, *p* < 0.05), while being significantly negatively correlated with *Sordariales* and *Mortierella* (|*r*| > 0.5, *p* < 0.05). The abundance of *M. oryzae* was significantly positively associated with *Bisifusarium* (|*r*| > 0.5, *p* < 0.05), but significantly negatively correlated with *Pseudeurotium* (|*r*| > 0.5, *p* < 0.05). Notably, *M. oryzae* was annotated as *Sordariomycetes*, which may be attributed to incomplete taxonomic coverage of the strain in the SILVA reference database, particularly for certain specialized strains or newly sequenced variants, resulting in classification resolution limited to the class level.

PICRUSt2 analysis predicted the functional abundance of soil microbiota involved in C- and N-cycling enzymes (Supplementary Figure S4). In S3 and S5, the urease abundance followed a trend consistent with measured enzyme activity and was significantly higher than in SCK (*p* < 0.05). Compared to SCK, S3 and S5 exhibited an increasing trend in β-xylosidase and NAG abundance, though the differences were not statistically significant (*p* > 0.05).

### 3.3 Straw retention enriches C cycling and N metabolic pathway genes

Metagenomic sequencing generated 553,203,650 raw reads across all treatments. Following normalization, we identified 2,996,412 reads matching 525 carbohydrate-active enzymes (CAZymes). The CAZy annotation identified six primary CAZy functional gene families: glycoside hydrolases (GHs), glycosyl transferases (GTs), polysaccharide lyases (PLs), carbohydrate esterases (CEs), carbohydrate-binding modules (CBMs), and auxiliary activities (AAs; Supplementary Figure S2). In S3 and S5, the abundance of GHs in the soil increased significantly (*p* < 0.05), whereas the abundance of GTs and PLs decreased significantly (*p* < 0.05).

Straw retention for 3 and 5 years significantly increased the absolute abundance of *sdimo* (*p* < 0.05; Supplementary Table S4). The S3 and S5 treatments significantly increased the absolute abundance of some soil functional genes associated with N cycling, such as nitrification-related genes (*amoA* and *amoB*) and N fixation-related genes (*nifH*), but significantly decreased the abundance of denitrification-related genes (*nirS*; *p* < 0.05).

**Figure 2:**
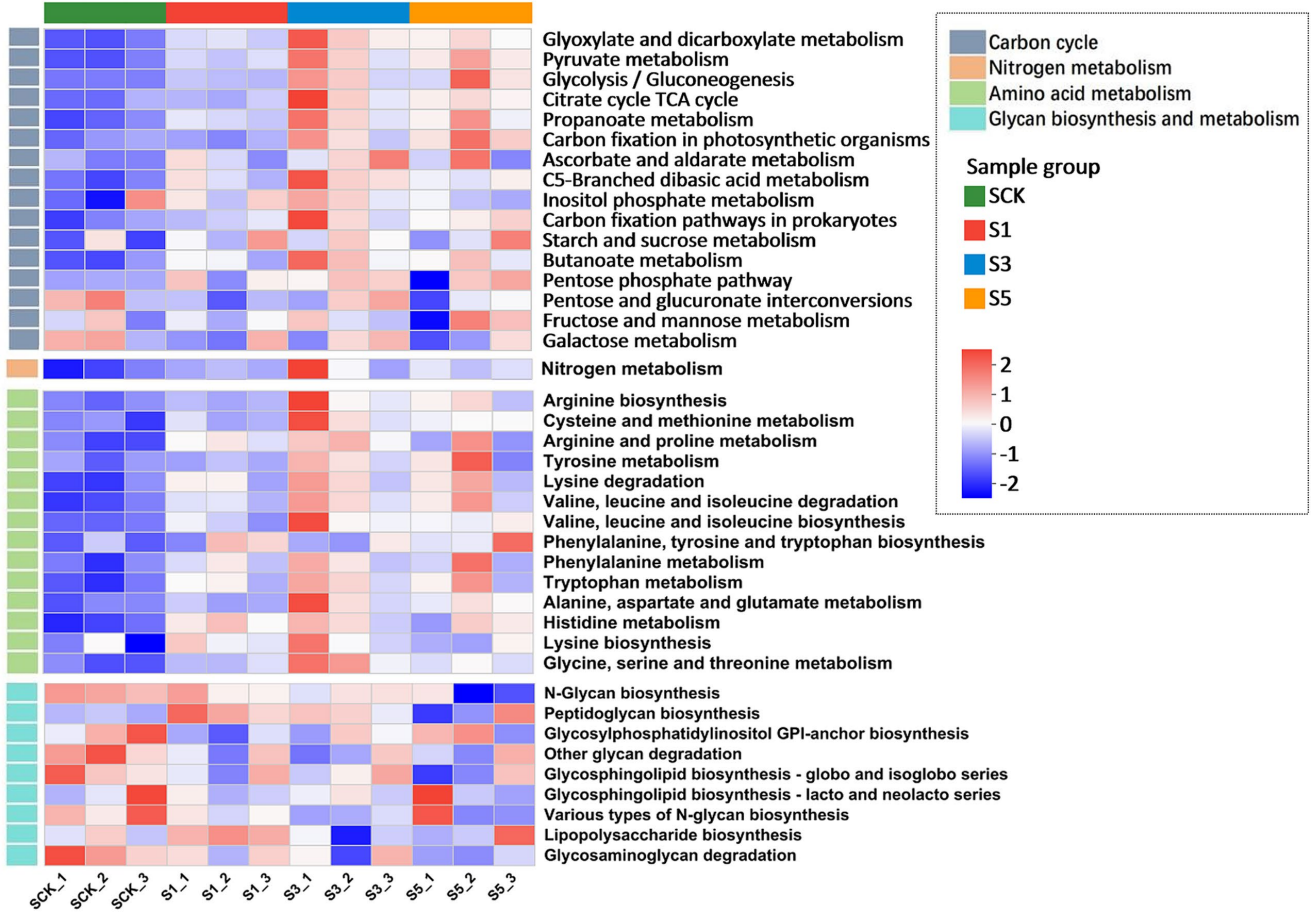
Heatmaps indicating differences in the relative abundances of functional genes related to the C and N cycles based on the Kyoto Encyclopedia of Genes and Genomes (KEGG) database. The relative abundances of the functional genes are indicated by the color intensity within the legend. The correlation coefficients ranging from negative to positive are indicated by color intensity changing from blue to red. SCK, the straw was not returned to the field; S1, S3, and S5, the straw was returned to the field consecutively for 1, 3, and 5 years, respectively.

**Figure 3:**
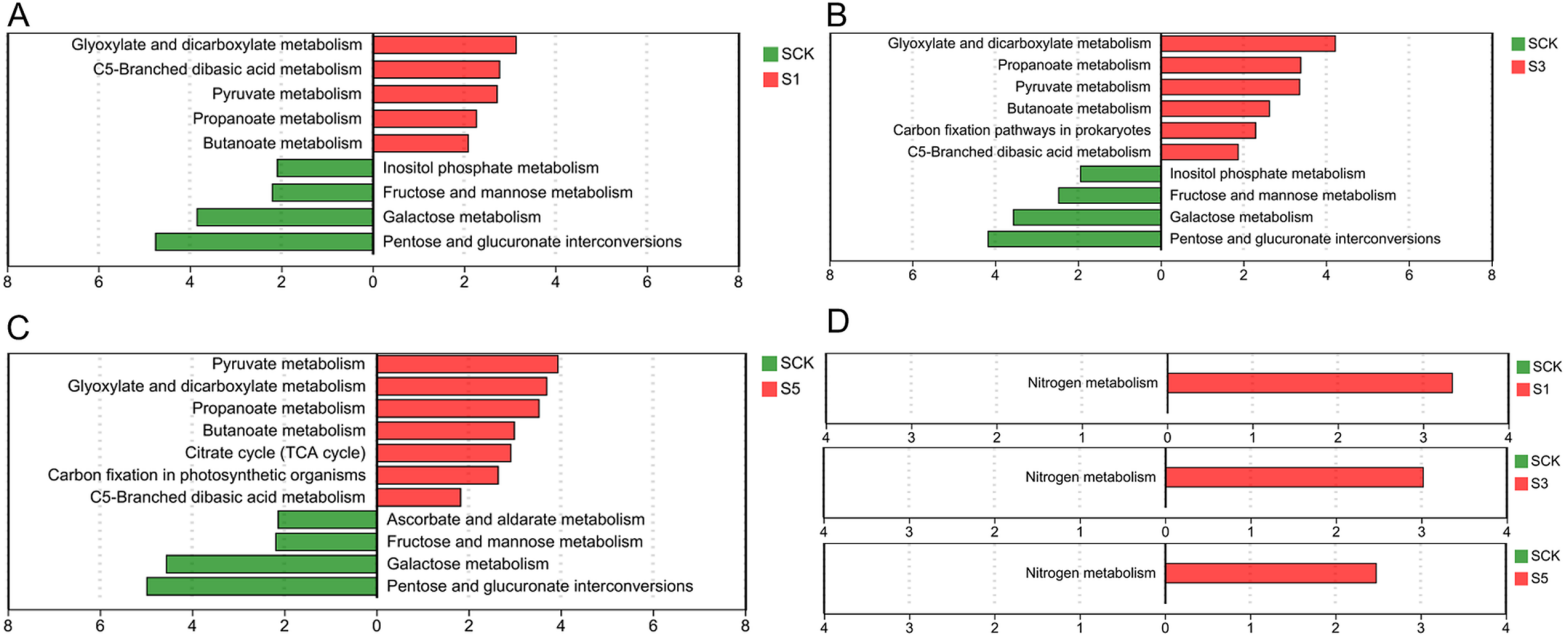
KEGG functional enrichment analysis. **(A–C)** Carbon cycling functional genes and **(D)** nitrogen metabolism genes. Green indicates genes enriched in the control group; red indicates genes enriched in the treatment group. The Student’s *t*-test was used to compare statistical differences between groups. The *p* value was corrected using a multiple test with false discovery rate (FDR). SCK, the straw was not returned to the field; S1, S3, and S5, the straw was returned to the field consecutively for 1, 3, and 5 years, respectively.

Metabolic pathway heat map analysis showed that straw retention stimulated the enrichment of genes involved in the C cycle, N metabolism, and amino acid metabolism (Figure 2). The results of the C cycling functional enrichment analysis showed that straw retention for 1 year (Figure 3A) increased the abundances of genes related to the metabolism of glyoxylate, dicarboxylate, C5-branched dibasic acid, pyruvate, propionate, and butanoate and decreased genes related to the metabolism of inositol phosphate, fructose, mannose, galactose, pentose, and glucuronate interconversions. Furthermore, straw retention for 3 years increased the abundance of genes related to C fixation pathways in prokaryotes (Figure 3B). Straw retention for 5 years increased the abundance of genes related to citrate (TCA) cycling and C fixation in photosynthetic organisms (Figure 3C).

**Figure 4:**
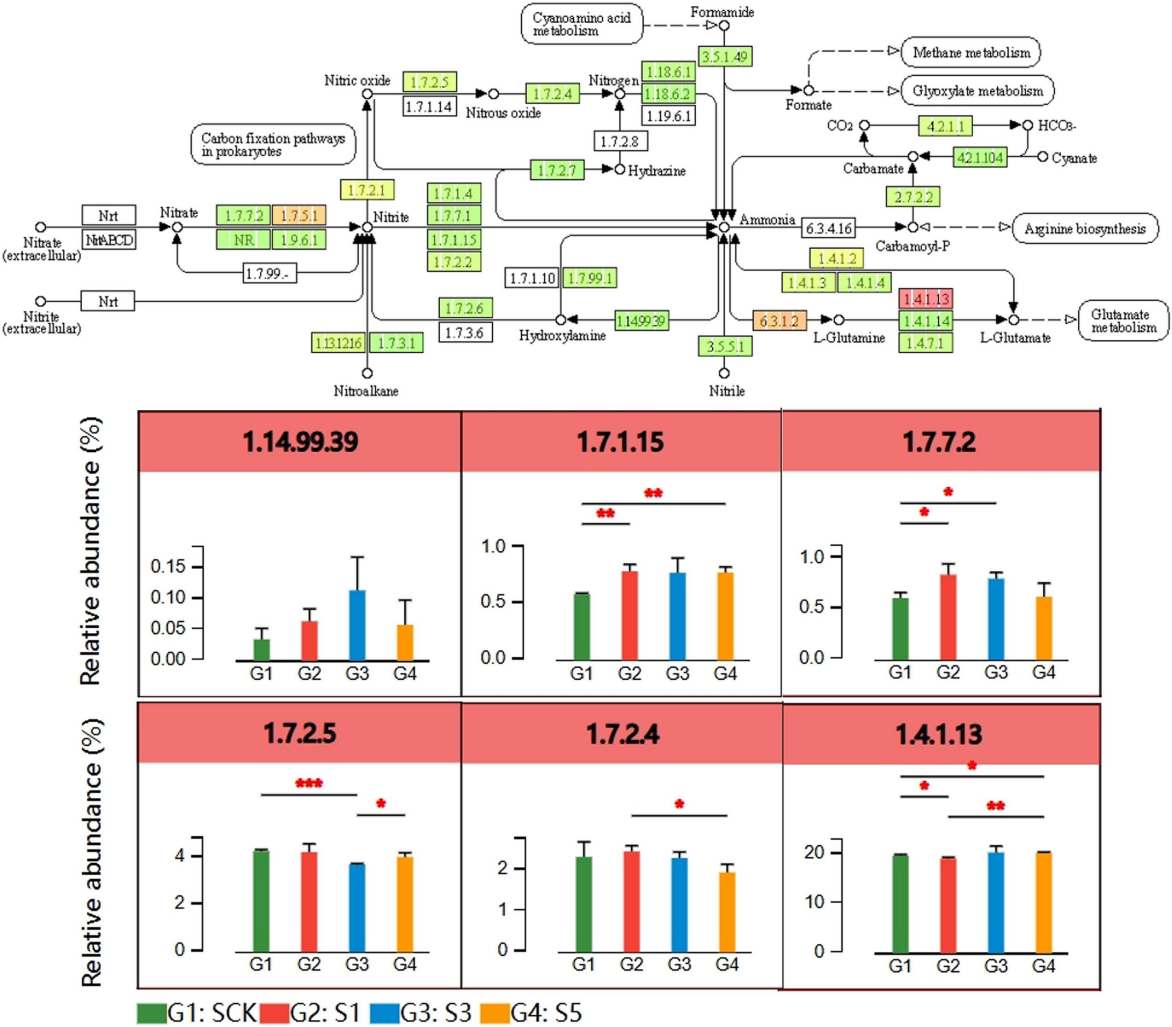
Analysis of differentially enriched KEGG pathways related to N metabolism (*p < 0.05; **p < 0.01; ***p < 0.001). The Student’s *t*-test was used to compare statistical differences between groups. The *p* value was corrected using a multiple test with false discovery rate (FDR). SCK, the straw was not returned to the field; S1, S3, and S5, the straw was returned to the field consecutively for 1, 3, and 5 years, respectively.

Functional enrichment analysis showed that straw retention enriched genes related to N metabolism (Figure 3D). Differentially enriched KEGG pathway analysis for N metabolism (Figure 4) showed that compared with the CK, dissimilatory nitrate reduction-related genes (*nirD*, *nirB* (1.7.1.15)) and assimilatory nitrate reduction-related genes (*narB* (1.7.7.2)) were significantly increased (*p* < 0.05) with straw retention. Conversely, straw retention decreased the abundance of denitrification-related pathway genes (*norB*, *norC* (1.7.2.5)). Furthermore, straw retention for 5 years enriched the abundance of L-asparaginase genes (*gltB*, *gltD* (1.4.1.13)), increasing the conversion between glutamine and glutamate with NH4^+^.

### 3.4 Correlations among the soil microbial communities, microbial functions, and soil physiochemical properties

**Figure 5:**
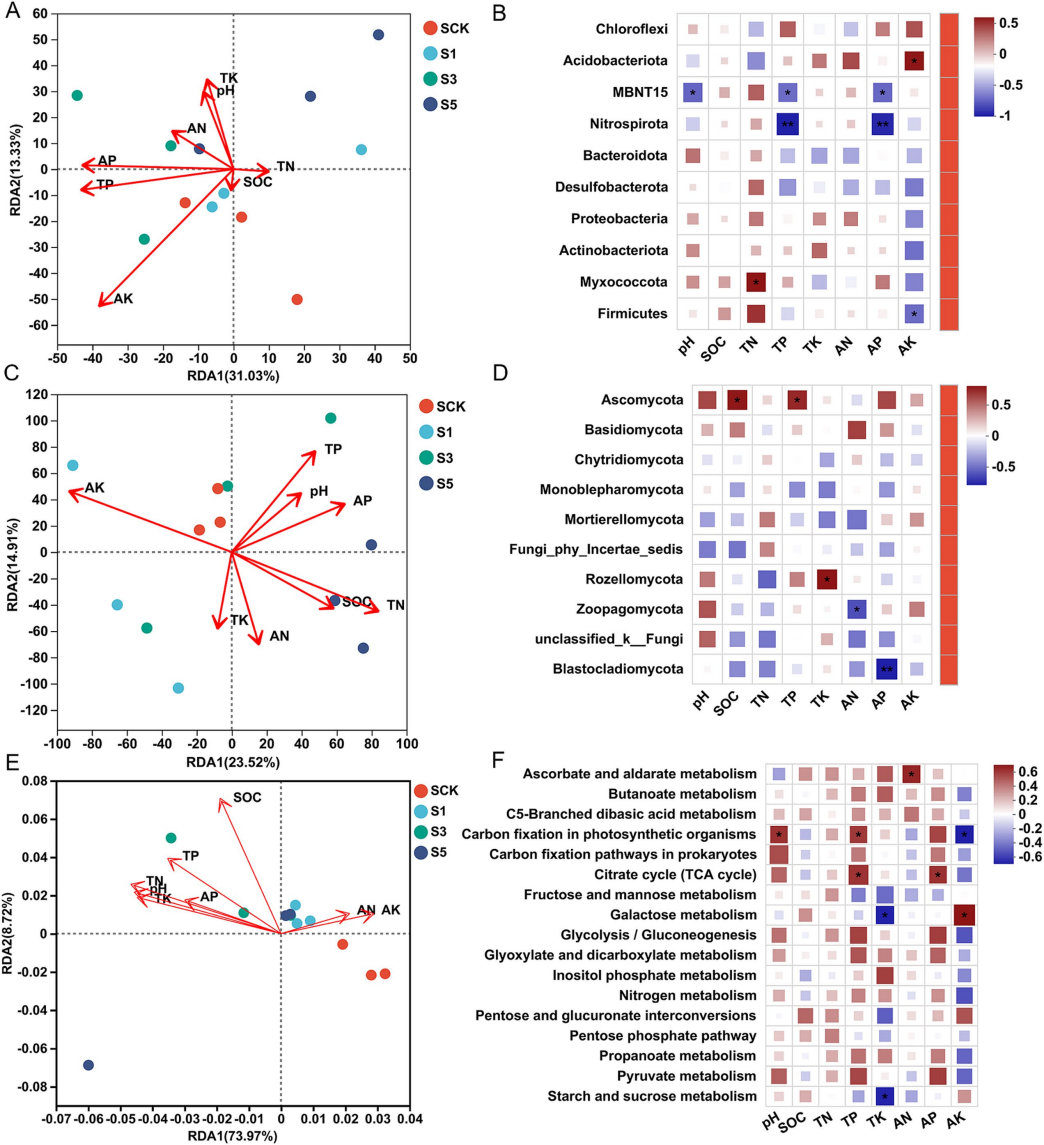
Redundancy analysis (RDA) and Spearman’s correlation analysis of soil physicochemical properties with bacteria **(A,D)**, fungal microbial communities **(B,E)**, and microbial functions **(C,F)**. The *p* value was corrected using a multiple test with false discovery rate (FDR). *p < 0.05 and **p < 0.01.

RDA was conducted to evaluate correlations among microbial communities, microbial functions, and soil physicochemical factors. AK significantly affected soil bacterial communities (*r* = 0.576; *p* = 0.026; Figure 5A), whereas soil TN (*r* = 0.509; *p* = 0.042) and AK (*r* = 0.465; *p* = 0.056) were positively correlated with the soil fungal communities (Figure 5B). SOC (*r* = 0.423; *p* = 0.122) was positively correlated with soil microbial C cycling and N metabolic pathways (Figure 5C).

Spearman’s correlation coefficient analysis revealed that soil TN was significantly positively correlated with the abundance of Myxococcota, and soil TP and AP were significantly negatively correlated with the abundance of Spirospirota. The soil AK was significantly positively correlated with Acidobacteriota but significantly negatively correlated with Firmicutes (Figure 5D).

Analysis of the soil fungi community showed that the soil SOC and TP significantly correlated with the abundance of Ascomycota. Soil TK was significantly positively correlated with the abundance of Rozellomycota, whereas soil AN was negatively correlated with the abundance of Zoopagomycota. A significant negative correlation was observed between soil AP and the abundance of Blastocladiomycota (Figure 5E).

Analysis of the microbial function pathways showed that soil pH, TP, and C fixation by photosynthetic organisms were significantly positively correlated. TP and AP were significantly positively correlated with the TCA cycle. Furthermore, TK was significantly negatively correlated with galactose, starch, and sucrose metabolism, and AN was significantly positively correlated with ascorbate and aldarate metabolism. Soil AK was significantly negatively correlated with carbon fixation in photosynthetic organisms but positively correlated with galactose metabolism (Figure 5F).

### 3.5 C cycle- and N metabolism-related taxa and function contribution analysis

**Figure 6:**
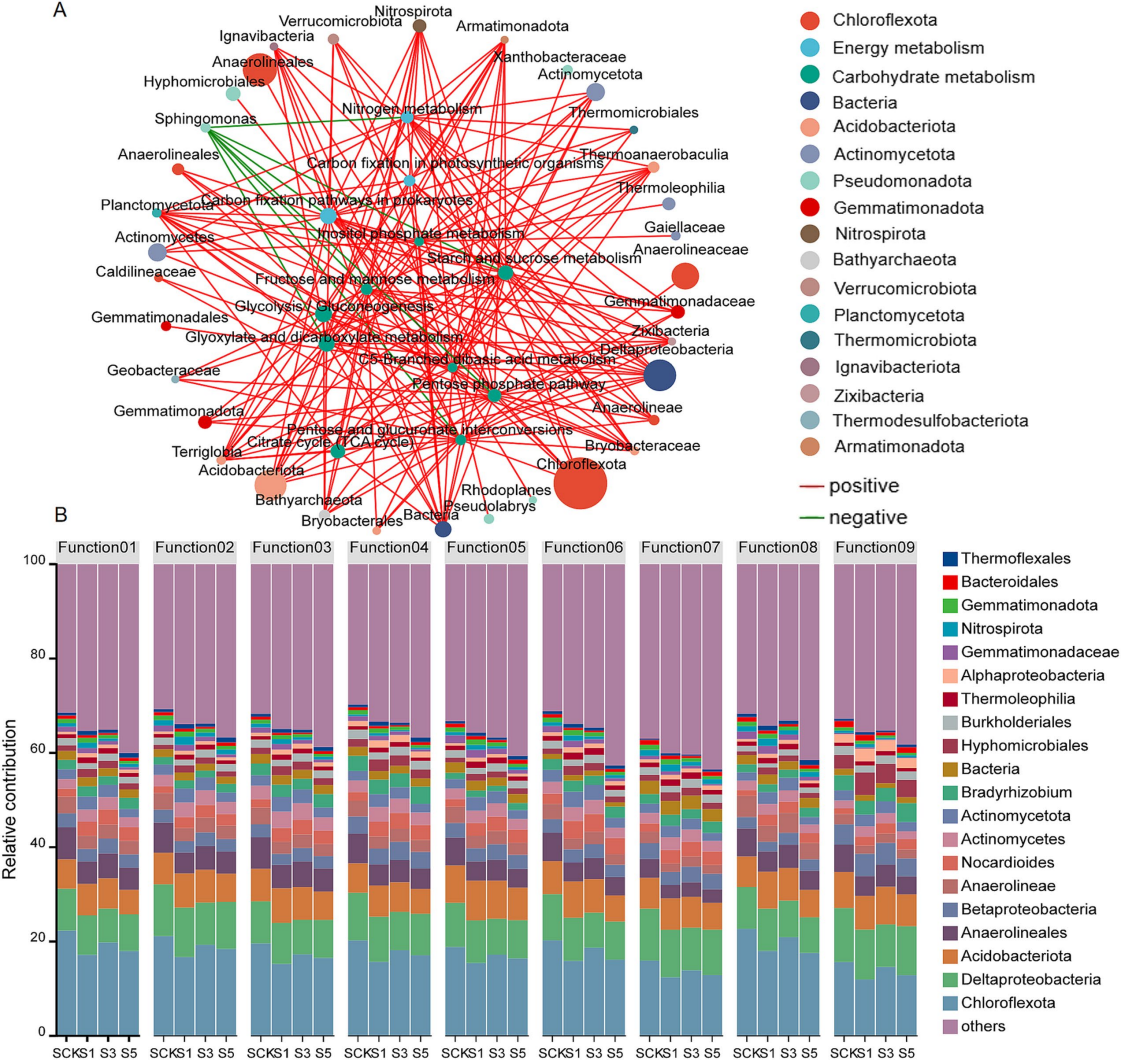
Correlation network analysis between species and the C-cycle, N-metabolism **(A)**, and functional contribution analysis **(B)**. Statistical correlations were examined through Spearman’s rank-order correlation analysis, with strong significant associations defined by |*r*| > 0.8 and *p* < 0.001. The *r* value is indicated by the color intensity within the legend. The correlation coefficients ranging from negative to positive are indicated by color intensity changing from blue to red. Function01: pyruvate metabolism; Function02: carbon fixation pathways in prokaryotes; Function03: glyoxylate and dicarboxylate metabolism; Function04: butanoate metabolism; Function05: citrate cycle (TCA cycle); Function06: propanoate metabolism; Function07: nitrogen metabolism; Function08: carbon fixation in photosynthetic organisms; Function09: C5-branched dibasic acid metabolism.

The correlations between the top 100 genera in terms of microbial abundance, C cycle, and N metabolism in the soil were evaluated using a correlation network diagram (|*r*| > 0.8; *p* < 0.001; Figure 6A). Thirty-three genera showed significant positive correlations with the C cycling pathway. Furthermore, *Planctomycetota*, *Deltaproteobacteria*, *Terriglobia*, *Armatimonadota, Thermoanaerobaculia*, *Acidobacteriota*, and *Nitrospirota* were significantly positively correlated with 10 or more C cycling pathways and N metabolic pathways, indicating that these bacteria form core functional microbiomes in the soil. In addition, g__*Sphingomonas* was significantly negatively correlated with seven C cycling and N metabolic pathways.

Species and functional contribution analysis revealed (Figure 6B) that the abundance of *Actinomycetes*, *Nocardioides*, *Actinomycetota*, *Bradyrhizobium*, *Hyphomicrobiales*, *Alphaproteobacteria*, and *Thermoflexales* increased in eight enhanced C cycling pathways. The abundance of *Nocardioides*, *Bradyrhizobium*, *Actinomycetota*, *Burkholderiales*, *Hyphomicrobiales*, and *Thermoleophilia* increased in the N metabolic pathways.

## 4 Discussion

### 4.1 Effects of straw retention on soil nutrient content

Straw decomposition releases nutrients such as C, N, P, and K, which enhance the availability of nutrients in the soil and increase soil fertility and organic matter content. Long-term, straw return with appropriate fertilizer can significantly enhance SOC, TN, AP, and AK (Su et al., 2020; Huang W. et al., 2021). In a previous study, short-term (5 years) straw return significantly enhanced soil SOC and AP levels (Xu et al., 2021). Similarly, SOC content was significantly increased when corn straw was returned to the field for two consecutive years (Fan and Wu, 2020), which is consistent with our findings. In this study, straw retention for 3 and 5 years significantly increased SOC and AP contents in the soil. These results indicate that continuous straw return rapidly increases SOC and AP. In contrast, increases in TK, TP, TN, AN, and AK levels were gradual and nonsignificant, even after 5 years of continuous straw return. SOC is recognized as a crucial indicator of soil fertility and significantly contributes to the sustainability of agroecosystems because of its effects on soil nutrient cycling, microbial properties, and physical structures (Liu et al., 2020; Peng et al., 2025).

### 4.2 Effects of straw retention on soil microbial communities

The soil microbial community, the most dynamic component of the soil microecosystem, is the vital biological indicator of soil quality and the most responsive factor to straw return (Liu et al., 2020). Straw retention increases microbial biomass by providing energy and nutrients for the growth of microbial communities (Sarker et al., 2019). Previous studies have found that short-term (3 years) crop residue return increases soil bacterial and fungal biomass (Chen et al., 2017). Long-term (14 and 30 years) straw return increases the soil fungal biomass but not bacterial biomass (Liu et al., 2022; Zhao et al., 2016). In this study, the bacterial biomass increased significantly, whereas the fungal biomass did not change significantly during the first year of straw retention. After 3 and 5 years of straw retention, the bacterial biomass reverted to the control levels, whereas the fungal biomass increased significantly. In the early stages of straw return, the presence of fresh, loosely structured plant residues with high water content attracts a large number of soil bacteria that facilitate the decomposition of straw. Initially, easily degradable components such as starch, hemicellulose, and cellulose are decomposed. In later stages, more recalcitrant compounds such as lignin are gradually broken down by soil fungi (Yu et al., 2020). With continuous straw return, the accumulation of lignin stimulates the growth of fungal biomass responsible for its decomposition.

Although 1 year of straw incorporation increased the absolute abundance of bacterial, it significantly decreased bacterial species richness. This reduction may be attributed to straw addition promoting the growth of cellulose-degrading bacteria, which occupied ecological niches and suppressed non-cellulose-degrading bacteria. With continuous straw incorporation, soil fungal diversity showed a significant decline by the fifth year. This phenomenon primarily resulted from the progressive accumulation of recalcitrant lignin in the soil under long-term straw application. This process selectively enriched lignin-degrading fungi, which gradually became the dominant decomposers, while the abundance of non-lignin-degrading fungi remained stable or decreased. Consequently, these changes led to a less even distribution of fungal species, ultimately reducing overall diversity.

The bacterial community composition underwent significant alterations. Straw retention for 5 years stimulated significant eutrophic bacterial growth, including Proteobacteria, Bacteroidota, and Firmicutes, which use labile forms of C for growth and metabolism and grow faster in nutrient-rich environments. The observed increase in bacterial growth can be attributed to enhanced soil fertility resulting from continuous straw retention. Bacteroidota and Firmicutes are fast-growing bacteria that exploit recalcitrant C sources (Huang et al., 2023). The continuous straw retention provided an abundant metabolic substrate for the growth of both Bacteroidota and Firmicutes. Moreover, compared with CK levels, straw retention tended to reduce the abundance of Nitrospirota, which could be due to their preference for lower C and N concentrations (Jin et al., 2020). Although straw return decreased the abundance of *Nitrospirae*, it significantly increased that of the N-fixing bacterium *Bradyrhizobium* and tended to decrease that of the denitrifying bacterium *Gemmatimonadetes*. This indicates that returning straw to the soil annually enhances N fixation and reduces soil denitrification, ultimately increasing TN content and reducing N loss from the soil.

For the fungal community, successive straw retention for 5 years significantly stimulated the growth of Ascomycota phylum and *Pseudeurotium*, *Pseudogymnoascus*, *Sordariales*, *Trichosporiella*, *Clavidisculum*, and *Schizothecium* genera. Ascomycota effectively degrades cellulose and lignocellulose, playing a crucial role in decomposing recalcitrant substrates (Ma et al., 2013). Compared to decomposed straw return, the abundance of Ascomycota increased with fresh straw return (Su et al., 2020). *Schizothecium* is a cellulose-degrading fungus that promotes the conversion of lignin and cellulose into humus (Liu et al., 2022). The combined application of straw and fertilizer enriched the *Schizothecium,* accelerating straw degradation (Zhang et al., 2021). Overall, continuous straw return promoted the growth and proliferation of cellulose-decomposing microbial groups.

Studies have used multitrophic ecological networks to confirm that keystone species, rather than overall microbial communities, dominate microbial stability, soil nutrient cycling function, and crop production (Fan et al., 2021; Xun et al., 2021). In this study, we identified key microbial communities related to soil C and N nutrient cycling in paddy ecosystems using correlation network, species, and functional contribution analyses. Thirty-three genera were positively correlated with C cycling and N metabolic pathways. These genera belong to Chloroflexota, Actinomycetota, Acidobacteriota, and Pseudomonadota. Moreover, *Sphingomonas* was negatively correlated with the C and N cycles. The increase in C cycling and N metabolic functions was attributed to the functional contributions of *Nocardioides*, *Actinomycetota*, *Bradyrhizobium*, *Hyphomicrobiales*, and *Thermoflexales*. These microorganisms are key to nutrient cycling in paddy soils.

### 4.3 Effects of straw retention on the nutrient cycling functions of soil microorganisms

Soil microorganisms play a crucial role in the mineralization and decomposition of organic matter by releasing extracellular enzymes and engaging in nutrient cycling and metabolic processes (Cui et al., 2018). An imbalance between the microbial growth environment and their stoichiometry is the primary driver of soil extracellular enzyme C decomposition (Zhou J. S. et al., 2024). Straw retention provides large amounts of C and N as substrate sources for soil enzymes, promoting enzyme activity and providing a shield against C and N loss via a buffering effect (Ning et al., 2020). In a recent study, the addition of maize straw was found to enhance the activities of BG, CBH, and dehydrogenase, as well as the abundances of the *GH48*, *cbhI*, and *cbbL* genes, indicating enhanced native SOC mineralization (Zhou G. P. et al., 2024). Another study found that maize straw return effectively improved the activities of urease, BG, and NAG (Ning et al., 2021), which is consistent with our findings. In this study, the continuous input of rice straw provided organic substrates for C cycling and N metabolic enzymes and significantly increased the activities of β-xylosidase, NAG, and urease; however, it had no significant effect on the activities of CBH and BG. This may be because CBH and BG are active during the initial stage of straw decomposition, whereas β-xylosidase is activated during the later stages (Huang et al., 2023).

Straw retention for 3 and 5 years promoted soil C decomposition and mineralization by enhancing the abundance of GH family genes involved in C fixation and carbohydrate metabolism. GHs are primary carbohydrate enzymes that facilitate the hydrolysis of glycoside bonds of complex sugars into carbohydrates (Wardman et al., 2022). A recent study showed that adding exogenous C can affect the abundance of microbial genes involved in C cycling, which typically play critical roles in SOC decomposition and CO_2_ fixation (Tang et al., 2021). A high C:N ratio increases the bacterial C fixation potential (Li et al., 2021; Wang et al., 2024). The input of maize straw into the soil stimulates the enrichment of soil microbial C-sequestration genes (Duan et al., 2021). Similarly, rice straw was a substrate with a high C:N ratio that enriched the genes involved in the CO_2_ fixation pathway.

Biological N fixation is a crucial ecological process. Approximately 24% of the total crop biomass in farmland ecosystems originates from N fixation by nonsymbiotic microorganisms. However, long-term fertilization management has significantly weakened the N-fixing capabilities of soil microorganisms in agricultural lands. Consequently, the soil environment can no longer actively select essential N-fixing bacteria, leading to an irreversible decline in the N-fixation capacity of the soil (Fan et al., 2019). In this study, the absolute abundance of N fixation genes was increased in the fertilizer-treated straw return. This approach may help mitigate the decline in the soil microbial N fixation function typically associated with fertilizer use alone. Moreover, genes related to nitrate reduction (dissimilation and assimilation) significantly increased with the high C:N ratio established after returning rice straw to the field, reducing the rate of nitrate N to ammonia N and its accumulation in the soil ecosystem. In addition, compared with the CK, straw retention for 5 years significantly increased the abundance of functional genes associated with nitrification and significantly decreased the abundance of functional genes associated with the denitrification pathway. This shows that the addition of straw significantly improves N availability and N fertilizer usage and reduces the risk of N loss in gaseous form from soil.

### 4.4 Limitations and future prospects

Straw return is an effective method for improving soil fertility and functional diversity; however, it has some disadvantages. In this study, straw retention for 3 and 5 years significantly enriched the abundances of *M. oryza* and *U. virens*, which may increase the risk of pathogen infection in the following seasons. These results are consistent with those of previous studies. For example, long-term (10 years) crop residue retention significantly increased the abundances of *Fusarium graminearum* and *Fusarium moniliforme*, increasing the risk of maize root rot (Wang H. H. et al., 2020). This phenomenon was attributed to the straw used being infected by pathogenic fungi. This hypothesis is supported by another study that showed that incorporating diseased straw led to significant increases in disease severity; conversely, adding healthy rice straw (no disease) resulted in no significant increase in disease severity (Zhu et al., 2014). In addition, crop straw can create a favorable environment and supply substantial amounts of organic C and nutrient substrates, promoting the proliferation of certain phytopathogenic fungi (Kerdraon et al., 2019). The return of decomposed straw reduced pathogenic fungal populations (Su et al., 2020), and straw combined with a microbial inoculant reduced the pathogen content (Liu H. L. et al., 2021). To avoid pathogen accumulation, diseased straws should be removed from the field, pretreated, or combined with a microbial inoculant before incorporation.

## 5 Conclusion

The findings suggest that rice straw return for a year has negligible effects on soil nutrient content, enzyme activity, microbial composition, and microbial function. However, continuous rice straw return for 3 and 5 years significantly increased SOC, AP, and fungal biomass contents and promoted the growth and proliferation of microbial communities involved in cellulose decomposition. Moreover, rice straw retention improved soil fertility and nutrient availability by enhancing soil enzyme activity, improving microbial structure, and enriching genes associated with C and N cycling. However, rice straw return for 3 and 5 consecutive years also significantly accumulated rice pathogenic fungi. The results indicate that continuous rice straw return is an effective strategy for improving the microecological environment and ecological function of soil. Further research will be required to devise an effective strategy to limit or avoid the accumulation of pathogenic fungi, thus ensuring safe and efficient straw return to the field.

## Data Availability

The datasets presented in this study can be found in online repositories. The names of the repository/repositories and accession number(s) can be found at: https://www.ncbi.nlm.nih.gov/, PRJNA1209629; https://www.ncbi.nlm.nih.gov/, PRJNA1209633; https://www.ncbi.nlm.nih.gov/, PRJNA121128.

## Footnotes

^1^ https://sourceforge.net/projects/flashpage/

^2^ http://drive5.com/uparse/

^3^ http://rdp.cme.msu.edu/

^4^ http://unite.ut.ee/index.php

^5^ http://www.arbsilva.de

^6^ http://www.illumina.com

^7^ http://www.majorbio.com

## References

[ref1] Bai X. Hu X. J. Liu J. J. Wei D. Zhu P. Cui X.. (2022). Ammonia oxidizing bacteria dominate soil nitrification under different fertilization regimes in black soils of Northeast China. Eur. J. Soil Biol. 111:103410. doi: 10.1016/j.ejsobi.2022.103410

[ref2] Chen Z. M. Wang H. Y. Liu X. W. Zhao X. L. Lu D. J. Zhou J. M.. (2017). Changes in soil microbial community and organic carbon fractions under short-term straw return in a rice-wheat cropping system. Soil Tillage Res. 165, 121–127. doi: 10.1016/j.still.2016.07.018

[ref3] Chen S. F. Zhou Y. Q. Chen Y. R. Gu J. (2018). fastp: an ultra-fast all-in-one FASTQ preprocessor. Bioinformatics 34, i884–i890. doi: 10.1093/bioinformatics/bty560, PMID: 30423086 PMC6129281

[ref4] Coban O. Deyn G. B. D. Ploeg M. J. (2022). Soil microbiota as game-changers in restoration of degraded lands. Science 375:abe0725. doi: 10.1126/science.abe0725, PMID: 35239372

[ref5] Cui Y. X. Fang L. C. Guo X. B. Wang X. Zhang Y. J. Li P.. (2018). Ecoenzymatic stoichiometry and microbial nutrientlimitation in rhizosphere soil in the arid area of the northern loess plateau, China. Soil Biol. Biochem. 116, 11–21. doi: 10.1016/j.soilbio.2017.09.025

[ref6] Cui H. Shutes B. Hou S. N. Wang X. Y. Zhu H. (2024). Long-term organic fertilization increases phosphorus content but reduces its release in soil aggregates. Appl. Soil Ecol. 203:105684. doi: 10.1016/j.apsoil.2024.105684

[ref7] Cui H. Zhu H. Shutes B. Rousseau A. N. Feng W. D. Hou S. N.. (2023). Soil aggregate-driven changes in nutrient redistribution and microbial communities after 10-year organic fertilization. J. Environ. Manag. 348:119306. doi: 10.1016/j.jenvman.2023.119306, PMID: 37839204

[ref8] Duan Y. Chen L. Li Y. M. Wang Q. Y. Zhang C. Z. Ma D. H.. (2021). N, P and straw return influence the accrual of organic carbon fractions and microbial traits in a Mollisol. Geoderma 403:115373. doi: 10.1016/j.geoderma.2021.115373

[ref9] Fan K. K. Delgado-Baquerizo M. Guo X. S. Wang D. Z. Wu Y. Y. Zhu M.. (2019). Suppressed N fixation and diazotrophs after four decades of fertilization. Microbiome 7:143. doi: 10.1186/s40168-019-0757-8, PMID: 31672173 PMC6824023

[ref10] Fan K. K. Delgado-Baquerizo M. Guo X. S. Wang D. Z. Zhu Y. G. Chu H. Y. (2021). Biodiversity of key-stone phylotypes determines crop production in a 4-decade fertilization experiment. ISME J. 15, 550–561. doi: 10.1038/s41396-020-00796-8, PMID: 33028975 PMC8027226

[ref11] Fan W. Wu J. G. (2020). Short-term effects of returning granulated straw on soil microbial community and organic carbon fractions in dryland farming. J. Microbiol. 58, 657–667. doi: 10.1007/s12275-020-9266-5, PMID: 32583286

[ref12] Huang J. J. Gao K. L. Yang L. Lu Y. H. (2023). Successional action of Bacteroidota and Firmicutes in decomposing straw polymers in a paddy soil. Environ. Microbiome 18:76. doi: 10.1186/s40793-023-00533-6, PMID: 37838745 PMC10576277

[ref13] Huang Y. P. Wang Q. Q. Zhang W. J. Zhu P. Xiao Q. Wang C. J.. (2021). Stoichiometric imbalance of soil carbon and nutrients drives microbial community structure under long-term fertilization. Appl. Soil Ecol. 168:104119. doi: 10.1016/j.apsoil.2021.104119

[ref14] Huang W. Wu J. F. Pan X. H. Tan X. M. Zeng Y. J. Shi Q. H.. (2021). Effects of long-term straw return on soil organic carbon fractions and enzyme activities in a double-cropped rice paddy in South China. J. Agricult. Sci. 20, 236–247. doi: 10.1016/S2095-3119(20)63347-0, PMID: 40322220

[ref16] Jin S. L. Jin W. Dong C. X. Bai Y. J. Jin D. C. Hu Z. J.. (2020). Effects of rice straw and rice straw ash on rice growth and α-diversity of bacterial community in rare-earth mining soils. Sci. Rep. 10:10331. doi: 10.1038/s41598-020-67160-w, PMID: 32587300 PMC7316728

[ref17] Kerdraon L. Laval V. Suffert F. (2019). Microbiomes and pathogen survival in crop residues, an ecotone between plant and soil. Phytobiomes J. 3, 246–255. doi: 10.1094/PBIOMES-02-19-0010-RVW

[ref18] Klimasmith I. M. Kent A. D. (2022). Micromanaging the nitrogen cycle in agroecosystems. Trends Microbiol. 30, 1045–1055. doi: 10.1016/j.tim.2022.04.006, PMID: 35618540

[ref19] Li Q. Kong B. H. Fan J. H. Cai H. Fu Y. Chen H. R. (2011). Early detection of rice blast by TaqMan real-time flourescence quantitative polymerase chain reaction. Acta Phytopathol. Sin. 41, 118–123. doi: 10.13926/j.cnki.apps.2011.02.002

[ref20] Li Z. W. Tong D. Nie X. D. Xiao H. B. Jiao P. P. Jiang J. Y.. (2021). New insight into soil carbon fixation rate: the intensive co-occurrence network of autotrophic bacteria increases the carbon fixation rate in depositional sites. Agric. Ecosyst. Environ. 320:107579. doi: 10.1016/j.agee.2021.107579

[ref21] Li Y. L. Tremblay J. L. Bainard L. D. Cade-Menun B. Hamel C. (2020). Long-term effects of nitrogen and phosphorus fertilization on soil microbial community structure and function under continuous wheat production. Environ. Microbiol. 22, 1066–1088. doi: 10.1111/1462-2920.14824, PMID: 31600863

[ref22] Lin J. Cheng Q. Kumar A. Zhang W. Yu Z. Hui D.. (2025). Effect of degradable microplastics, biochar and their coexistence on soil organic matter decomposition: a critical review. TrAC Trends Anal. Chem. 183:118082. doi: 10.1016/j.trac.2024.118082

[ref23] Liu W. J. Graham E. B. Zhong L. H. Zhang J. W. Li W. T. Li Z. P.. (2020). Dynamic microbial assembly processes correspond to soil fertility in sustainable paddy agroecosystems. Funct. Ecol. 34, 1244–1256. doi: 10.1111/1365-2435.13550, PMID: 40304126

[ref24] Liu H. L. Qi Y. Q. Wang J. H. Jiang Y. Geng M. X. (2021). Synergistic effects of crop residue and microbial inoculant on soil properties and soil disease resistance in a Chinese Mollisol. Sci. Rep. 11:24225. doi: 10.1038/s41598-021-03799-3, PMID: 34930990 PMC8688499

[ref25] Liu J. A. Shu A. P. Song W. F. Shi W. C. Li M. C. Zhang W. X.. (2021). Long-term organic fertilizer substitution increases rice yield by improving soil properties and regulating soil bacteria. Geoderma 404, 115287–115296. doi: 10.1016/j.geoderma.2021.115287

[ref26] Liu B. Xia H. Jiang C. C. Riaz M. Yang L. Chen Y. F.. (2022). 14 year applications of chemical fertilizers and crop straw effects on soil labile organic carbon fractions, enzyme activities and microbial community in rice-wheat rotation of middle China. Sci. Total Environ. 841:156608. doi: 10.1016/j.scitotenv.2022.156608, PMID: 35700778

[ref27] Ma A. Z. Zhuang X. L. Wu J. M. Cui M. M. Lv D. Liu C. Z.. (2013). Ascomycota members dominate fungal communities during straw residue decomposition in arable soil. PLoS One 8, 1475–1478. doi: 10.1371/journal.pone.0066146, PMID: 23840414 PMC3688710

[ref28] Ning X. L. Wang X. H. Guan Z. Y. Gu Y. Wu C. S. Hu W. H. (2021). Effects of different patterns of maize-straw application on soil microorganisms, enzyme activities, and grain yield. Bioengineered 12, 3684–3698. doi: 10.1080/21655979.2021.1931639, PMID: 34254569 PMC8806571

[ref29] Ning Y. F. Wei L. Wei X. M. Zhu Z. K. Wu J. S. (2020). Response of extracellular enzyme activities to substrate availability in paddy soil with long-term fertilizer management. Huan Jing Ke Xue 41, 2852–2860. doi: 10.13227/j.hjkx.201909140, PMID: 32608802

[ref30] Pang Q. Zhao G. Wang D. Zhu X. Xie L. Zuo D.. (2024). Water periods impact the structure and metabolic potential of the nitrogen-cycling microbial communities in rivers of arid and semi-arid regions. Water Res. 267:122472. doi: 10.1016/j.watres.2024.122472, PMID: 39305525

[ref31] Peng C. Xu W. Wang X. Meng F. Zhao Y. Wang Q.. (2025). Alginate oligosaccharides trigger multiple defence responses in tobacco and induce resistance to *Phytophthora infestans*. Front. Plant Sci. 16:1506873. doi: 10.3389/fpls.2025.1506873, PMID: 40012726 PMC11863610

[ref32] Sarker J. R. Singh B. P. Fang Y. Y. Cowie A. L. Dougherty W. J. Collins D.. (2019). Tillage history and crop residue input enhanced native carbon mineralisation and nutrient supply in contrasting soils under long-term farming systems. Soil Tillage Res. 193, 71–84. doi: 10.1016/j.still.2019.05.027

[ref33] Su Y. Lv J. Yu M. Ma Z. H. Xi H. Kou C.. (2020). Long-term of decomposed straw return positively affects the soil microbial community. J. Appl. Microbiol. 128, 138–150. doi: 10.1111/jam.14435, PMID: 31495045

[ref34] Sun X. Y. Kang S. Zhang Y. J. Tan X. Q. Yu Y. F. He H. Y.. (2013). Genetic diversity and population structure of rice pathogen Ustilaginoidea virens in China. PLoS One 8:e76879. doi: 10.1371/journal.pone.0076879, PMID: 24098811 PMC3786968

[ref35] Tang H. M. Li C. Xu Y. L. Cheng K. K. Shi L. H. Wen L.. (2021). Effects of fertilizer practice on fungal and actinobacterial cellulolytic community with different humified particle-size fractions in double-cropping field. Sci. Rep. 11:18441. doi: 10.1038/s41598-021-97975-0, PMID: 34531457 PMC8446020

[ref36] Tang Q. Xia Y. Q. Ti C. P. Shan J. Zhou W. Li C. L.. (2023). Partial organic fertilizer substitution promotes soil multifunctionality by increasing microbial community diversity and complexity. Pedosphere 33, 407–420. doi: 10.1016/j.pedsph.2022.06.044

[ref37] Wang Y. F. Chen P. Wang F. H. Han W. X. Qiao M. Dong W. X.. (2022). The ecological clusters of soil organisms drive the ecosystem multifunctionality under long-term fertilization. Environ. Int. 161:107133. doi: 10.1016/j.envint.2022.107133, PMID: 35149447

[ref38] Wang H. H. Li X. Li X. Wang J. Li X. Y. Guo Q. C.. (2020). Long-term no-tillage and different residue amounts alter soil microbial community composition and increase the risk of maize root rot in Northeast China. Soil Tillage Res. 196:104452. doi: 10.1016/j.still.2019.104452

[ref39] Wang C. Liu D. W. Bai E. (2018). Decreasing soil microbial diversity is associated with decreasing microbial biomass under nitrogen addition. Soil Biol. Biochem. 120, 126–133. doi: 10.1016/j.soilbio.2018.02.003

[ref40] Wang Y. J. Liu L. Yang J. F. Duan Y. M. Luo Y. Taherzadeh M. J.. (2020). The diversity of microbial community and function varied in response to different agricultural residues composting. Sci. Total Environ. 715:136983. doi: 10.1016/j.scitotenv.2020.136983, PMID: 32041001

[ref41] Wang Y. Lyu H. Du Y. Cheng Q. Liu Y. Ma J.. (2024). Unraveling how Fe-Mn modified biochar mitigates sulfamonomethoxine in soil water: the activated biodegradation and hydroxyl radicals formation. J. Hazard. Mater. 465:133490. doi: 10.1016/j.jhazmat.2024.133490, PMID: 38228002

[ref42] Wardman J. F. Bains R. K. Rahfeld P. Withers S. G. (2022). Carbohydrate-active enzymes (CAZymes) in the gut microbiome. Nat. Rev. Microbiol. 20, 542–556. doi: 10.1038/s41579-022-00712-1, PMID: 35347288

[ref43] Xu G. H. Chen X. M. Mao W. Li W. X. Yu J. Xia W. (2021). Effect of continuous Total amount straw returning to field on soil properties and Rice yield. Mod. Agricult. Sci. Technol. 23, 1–3+7. doi: 10.3969/j.issn.1007-5739.2021.23.001

[ref44] Xu Z. Y. Sun R. H. He T. Y. Sun Y. Z. Wu M. C. Xue Y. H.. (2023). Disentangling the impact of straw incorporation on soil microbial communities: enhanced network complexity and ecological stochasticity. Sci. Tot. Environ. 863:160918. doi: 10.1016/j.scitotenv.2022.160918, PMID: 36528952

[ref45] Xun W. B. Liu Y. P. Li W. Ren Y. Xiong W. Xu Z. H.. (2021). Specialized metabolic functions of keystone taxa sustain soil microbiome stability. Microbiome 9:35. doi: 10.1186/s40168-020-00985-9, PMID: 33517892 PMC7849160

[ref46] Yang Y. Chen X. L. Liu L. X. Li T. Dou Y. X. Qiao J. B.. (2022). Nitrogen fertilization weakens the linkage between soil carbon and microbial diversity: a global meta-analysis. Glob. Chang. Biol. 28, 6446–6461. doi: 10.1111/gcb.16361, PMID: 35971768

[ref47] Yang J. Q. Diao H. J. Hu S. Y. Wang C. H. (2021). Effects of nitrogen addition at different levels on soil microorganisms in saline-alkaline grassland of northern China. Chinese J. Plant Ecol. 45, 780–789. doi: 10.17521/cjpe.2021.0072

[ref48] Yang Y. Gunina A. Cheng H. Liu L. Wang B. Dou Y.. (2025). Unlocking mechanisms for soil organic matter accumulation: carbon use efficiency and microbial Necromass as the keys. Glob. Chang. Biol. 31:e70033. doi: 10.1111/gcb.70033, PMID: 39825463

[ref49] Yang X. Xia X. Zhang Z. Nong B. Zeng Y. Wu Y.. (2019). Identification of anthocyanin biosynthesis genes in rice pericarp using PCAMP. Plant Biotechnol. J. 17, 1700–1702. doi: 10.1111/pbi.13133, PMID: 31004548 PMC6686123

[ref50] Yin Y. Gong H. Chen Z. Tian X. Wang Y. Wang Z.. (2025). Underestimated sequestration of soil organic carbon in China. Environ. Chem. Lett. 23, 373–379. doi: 10.1007/s10311-024-01813-4

[ref51] Yu C. Li Y. Mo R. L. Deng W. Zhu Z. X. Liu D. B.. (2020). Effects of long-term straw retention on soil microorganisms under a rice-wheat cropping system. Arch. Microbiol. 202, 1915–1927. doi: 10.1007/s00203-020-01899-8, PMID: 32451591

[ref52] Yuan L. H. Gao Y. Mei Y. Liu J. R. Kalkhajeh Y. K. Hu H. X.. (2023). Effects of continuous straw returning on bacterial community structure and enzyme activities in rape-rice soil aggregates. Sci. Rep. 13:2357. doi: 10.1038/s41598-023-28747-1, PMID: 36759519 PMC9911641

[ref53] Yuan A. Kumar S. D. Wang H. Wang S. Impa S. Wang H.. (2024). Dynamic interplay among soil nutrients, rhizosphere metabolites, and microbes shape drought and heat stress responses in summer maize. Soil Biol. Biochem. 191:109357. doi: 10.1016/j.soilbio.2024.109357

[ref54] Zhang C. F. Lin Z. L. Que Y. X. Fallah F. Tayyab M. Li S. Y.. (2021). Straw retention efficiently improves fungal communities and functions in the fallow ecosystem. BMC Microbiol. 21:52. doi: 10.1186/s12866-021-02115-3, PMID: 33596827 PMC7890633

[ref55] Zhang T. Song B. Han G. Zhao H. Hu Q. Zhao Y.. (2023). Effects of coastal wetland reclamation on soil organic carbon, total nitrogen, and total phosphorus in China: a meta-analysis. Land Degrad. Dev. 34, 3340–3349. doi: 10.1002/ldr.4687

[ref56] Zhao S. C. Li K. J. Zhou W. Qiu S. J. Huang S. W. He P. (2016). Changes in soil microbial community, enzyme activities and organic matter fractions under long-term straw return in north Central China. Agric. Ecosyst. Environ. 216, 82–88. doi: 10.1016/j.agee.2015.09.028

[ref57] Zhou G. P. Fan K. K. Gao S. J. Chang D. Li G. L. Liang T.. (2024). Green manuring relocates microbiomes in driving the soil functionality of nitrogen cycling to obtain preferable grain yields in thirty years. Sci. China Life Sci. 67, 596–610. doi: 10.1007/s11427-023-2432-9, PMID: 38057623

[ref58] Zhou J. S. Zhang S. B. Lv J. Y. Tang C. X. Zhang H. B. Fang Y. Y.. (2024). Maize straw increases while its biochar decreases native organic carbon mineralization in a subtropical forest soil. Sci. Total Environ. 939:173606. doi: 10.1016/j.scitotenv.2024.173606, PMID: 38823704

[ref59] Zhu H. Wang Z. Luo X. Song J. X. Huang B. (2014). Effects of straw incorporation on Rhizoctonia solani inoculum in paddy soil and rice sheath blight severity. J. Agric. Sci. 152, 741–748. doi: 10.1017/S002185961300035X

