# Supplementary material for: Straw retention drives microbial community succession to improve soil C/N cycling: insights from a multi-year rice-based system
Source: Front Microbiol. 2025 May 20;16:1590788. doi: 10.3389/fmicb.2025.1590788 (PMC12129795; doi:10.3389/fmicb.2025.1590788)
Supplement: Supplementary file 1 [file Data_Sheet_1.docx]

Supplementary Material

**Altering microbial community for improving soil properties and carbon and nitrogen cycling through rice straw retention: Insights from a multiannual study in Northeast China**

**Shu Jia^1^, Yue-dong Li^2^, Hang Qu^1^, Bo Li, Ying-hua Juan^1^, Yue-hua Xing^1^, Yan Liu^1^, and Hong-jing Bao^1^, Wen-tao Sun^1*^**

*** Correspondence:**

Wen-tao Sun: [wentaosw@163.com](mailto:wentaosw@163.com)

# Supplementary Figures and Tables

## Supplementary Figures


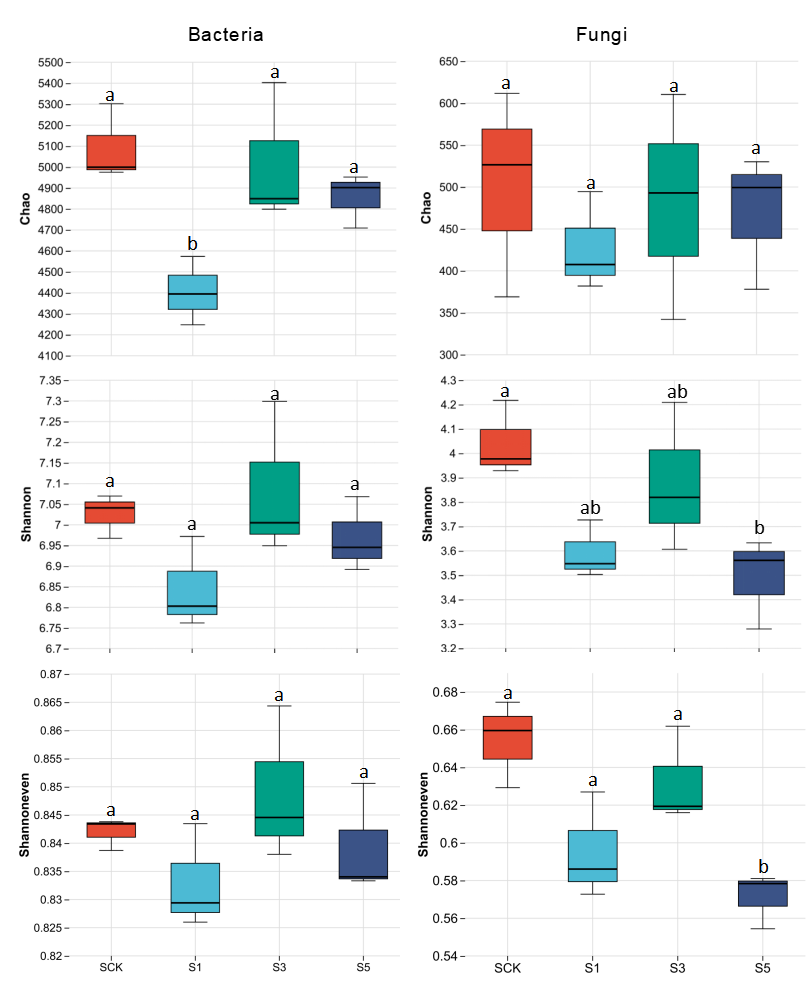


**Supplementary Figure 1.** Boxplots of bacterial and fungal diversity, richness and evenness (number of operational taxonomic units [OTUs]) detected in different treatments. The top and bottom whiskers indicate the maximum and minimum values, respectively, and the hyphen represents the median value. Values by different lowercase letters mean significant difference at *P* < 0.05 (one-way analysis of variance). The *P* value was corrected using a multiple test with false discovery rate (FDR). SCK, the straw was not returned to the field; S1, S3, and S5, the straw was returned to the field consecutively for 1, 3, and 5 years, respectively.

#
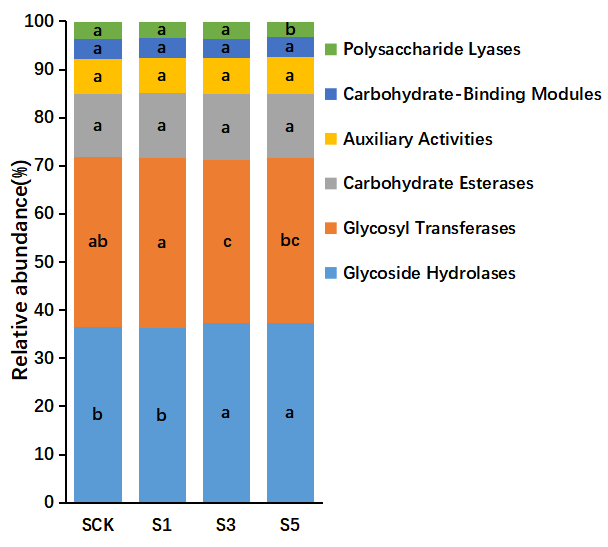


**Supplementary Figure 2.** Functional composition and relative abundance of primary functional layer bacteria based on Carbohydrate-Active Enzyme (CAZy) databases. Values different lower case letters indicate differed significantly at *P* < 0.05 (one-way analysis of variance). The *P* value was corrected using a multiple test with false discovery rate (FDR). SCK, the straw was not returned to the field; S1, S3, and S5, the straw was returned to the field consecutively for 1, 3, and 5 years, respectively.


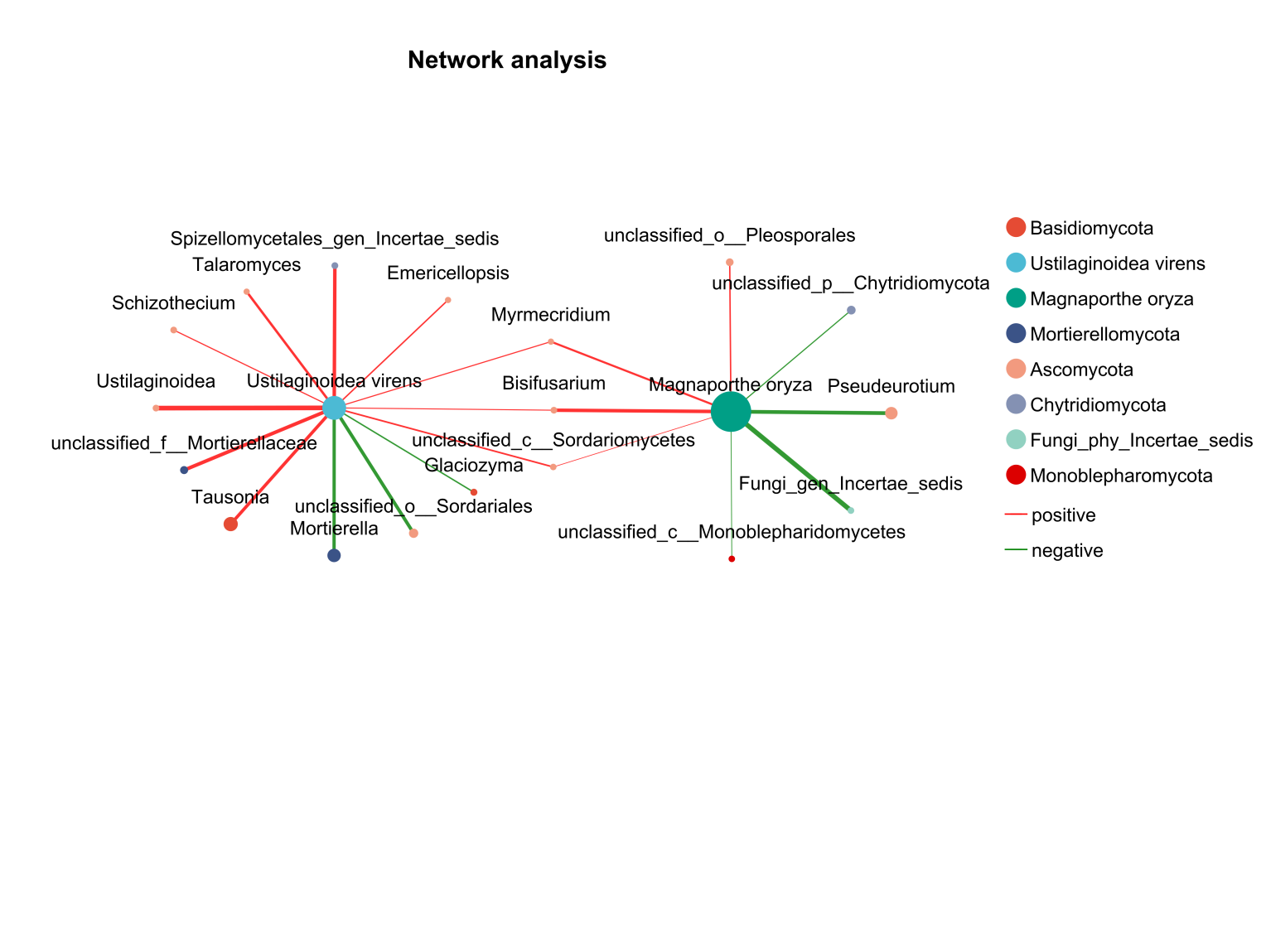


**Supplementary Figure 3**. Correlation network analysis between fungi species and *Magnaporthe oryza* and *Ustilaginoidea virens*. Statistical correlations were examined through Spearman's rank-order correlation analysis, with strong significant associations defined by |r| > 0.4. The color of the connecting lines indicates the direction of correlation: red lines represent positive correlations, while green lines denote negative correlations.


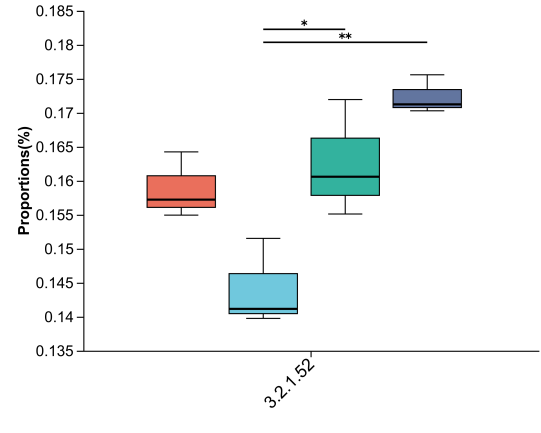

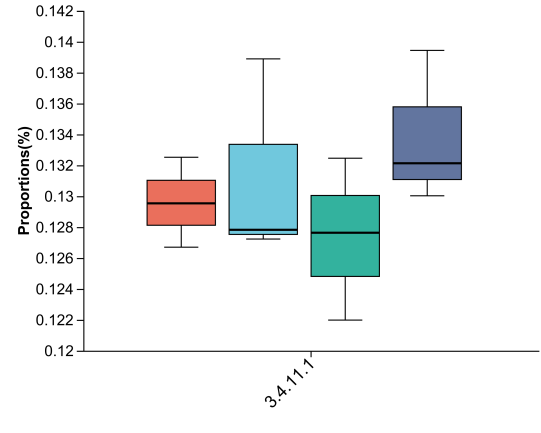

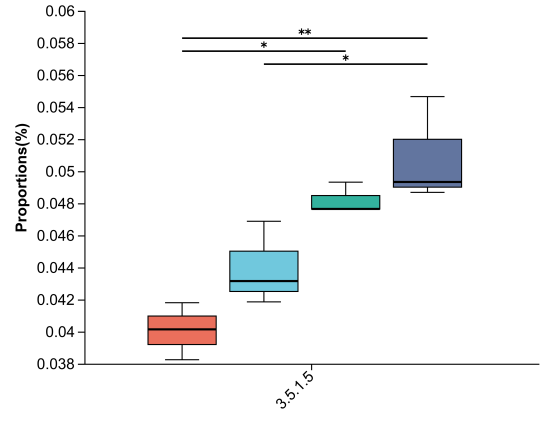

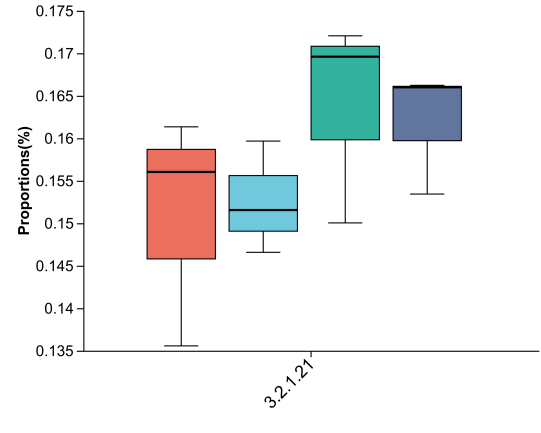

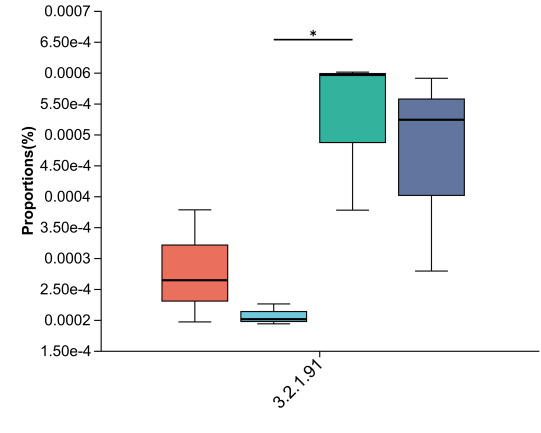

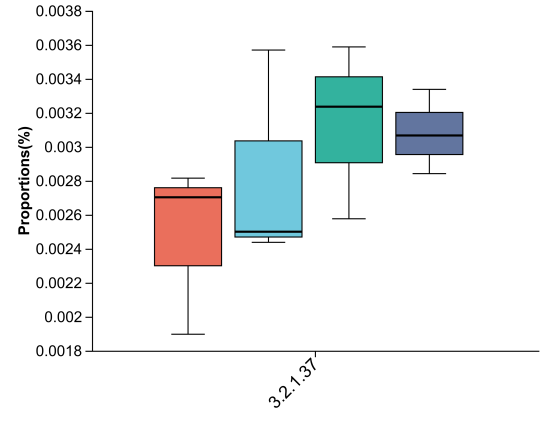

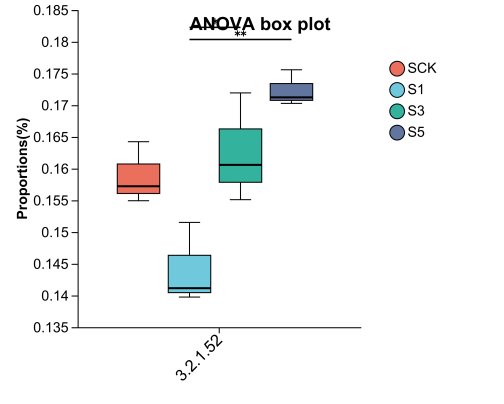


**Supplementary Figure 4**. PICRUSt2 functional Prediction of C/N-Cycling Enzymes in Bacterial Communities based on the KEGG pathway. The top and bottom whiskers indicate the maximum and minimum values, respectively, and the hyphen represents the median value. Values by different lowercase letters mean significant difference at *P* < 0.05 (one-way analysis of variance). The P value was corrected using a multiple test with false discovery rate (FDR). 3.2.1.21: β-glucosidase, 3.2.1.91: β-cellobiohydrolase, 3.2.1.37: β-xylosidase, 3.2.1.52: N-acetyl-glucosaminidase, 3.4.11.1: L-leucine aminopeptidase, 3.5.1.5: urease. SCK, the straw was not returned to the field; S1, S3, and S5, the straw was returned to the field consecutively for 1, 3, and 5 years, respectively.

# 1.2 Supplementary Tables

**Supplementary Table 1.** Soil physicochemical properties under continuous straw return for 1, 3, and 5 years

| **Treatment** | **pH** | **SOC(g/kg)** | **TN**（g/kg) | **TP**（g/kg) | **TK**（g/kg) | **AN**（mg/kg) | **AP**（mg/kg) | **AK**（mg/kg) |
| --- | --- | --- | --- | --- | --- | --- | --- | --- |
| SCK | 6.83±0.38a | 23.12±0.36b | 1.99±0.05a | 0.99±0.01a | 21.22±0.32a | 126.64±9.64a | 44.07±2.91bc | 210.33±23.17a |
| S1 | 6.76±0.19a | 23.96±0.29b | 2.03±0.09a | 0.96±0.01a | 21.59±0.20a | 130.84±12.1a | 35.87±2.43c | 210.00±8.89a |
| S3 | 7.06±0.18a | 25.95±0.69a | 1.96±0.23a | 1.18±0.07a | 21.42±0.12a | 132.07±11.07a | 63.50±10.29a | 221.67±9.74a |
| S5 | 7.06±0.17a | 24.79±0.17a | 2.06±0.01a | 1.01±0.01a | 21.46±0.09a | 130.53±4.22a | 53.03±1.68ab | 216.67±4.33a |
| Note: Data are presented as x±s( n=3). Values at the same column followed by different lower case letters differed significantly at P < 0.05 (one-way analysis of variance). SOC, Soil organic carbon; TN, Total nitrogen; TP, Total phosphorus; TK, Total potassium；AN, Available nitrogen; AP, Available phosphorous; AK, Available potassium. SCK, the straw was not returned to the field; S1, S3, and S5, the straw was returned to the field consecutively for 1, 3, and 5 years, respectively.Values different lower case letters indicate differed significantly at *P* < 0.05 (one-way analysis of variance). | | | | | | | | |

**Supplementary Table 2.** Sequences of primers and PCR conditions used in this study

| **Target** | **Primer name** | **Sequence(5′-3′)** | **PCR conditions** | **References** |
| --- | --- | --- | --- | --- |
| 16S rRNA | Eub338 | ACTCCTACGGGAGGCAGCAG | 35 cycles × (95 ℃, 30 s; 59 ℃, 30 s; 72 ℃,45 s) | Yang et al., 2021 |
|  | Eub806 | GGACTACHVGGGTWTCTAAT |  |  |
| ITS rRNA | ITS1F | CTTGGTCATTTAGAGGAAGTAA | 35 cycles × (95 ℃, 30 s; 59 ℃, 30 s; 72 ℃,45 s) |  |
|  | ITS2R | GCTGCGTTCTTCATCGATGC |  |  |
| 16S v3-v4 | 338F | ACTCCTACGGGAGGCAGCAG | 35 cycles × (95 ℃, 30 s; 59 ℃, 30 s; 72 ℃,45 s) |  |
|  | 806R | GGACTACHVGGGTWTCTAAT |  |  |
| ITS | ITS1 | CTTGGTCATTTAGAGGAAGTAA | 35 cycles × (95 ℃, 30 s; 59 ℃, 30 s; 72 ℃,45 s) |  |
|  | ITS2 | GCTGCGTTCTTCATCGATGC |  |  |
| *cbh I* | fungcbh IF | ACCAAYTGCTAYACIRGYAA | 35 cycles × (95 ℃, 30 s; 59 ℃, 30 s; 72 ℃,45 s) | Zhou et al., 2024a |
|  | fungcbh IR | GCYTCCCAIATRTCCATC |  |  |
| *GH48* | GH48_F8 | GCCADGHTBGGCGACTACCT | 40 cycles × (95 ℃, 5 s; 57 ℃, 20 s; 72 ℃,30 s) |  |
|  | GH48_R5 | CGCCCCABGMSWWGTACCA­­ |  |  |
| *sdimo* | NVC66 | CCANCCNGGRTAYTTRTTYTCRAACCA | 40 cycles × (95 ℃, 30 s; 53 ℃, 30 s; 72 ℃,45 s) |  |
|  | NVC57 | CAGTCNGAYGARKCSCGNCAYAT |  |  |
| *nifH* | nifH-F | AAAGGYGGWATCGGYAARTCCACCAC | 40 cycles × (95 ℃, 5 s; 55 ℃, 30 s; 72 ℃, 60 s) |  |
|  | nifH-R | TTGTTSGCSGCRTACATSGCCATCAT |  |  |
| *amoA* | Arch-amoA26F | GACTACATMTTCTAYACWGAYTGGGC | 40 cycles × (95 ℃, 30 s; 55 ℃, 30 s; 72 ℃,45 s) |  |
|  | Arch-amoA417R | GGKGTCATRTATGGWGGYAAYGTTGG |  |  |
| *amoB* | amoB-1F | GGGGTTTCTACTGGTGGT | 40 cycles × (95 ℃, 30 s; 54 ℃, 30 s; 72 ℃,45 s) |  |
|  | amoB-2R | CCCCTCKGSAAAGCCTTCTTC |  |  |
| *narG* | narG-1960m2F | TAYGTSGGGCAGGARAAACTG | 35 cycles × (95 ℃, 30 s; 56 ℃, 30 s; 72 ℃,45 s) |  |
|  | narG-2050m2R | CGTAGAAGAAGCTGGTGCTGTT |  |  |
| *nirS* | cd3aF | GTSAACGTSAAGGARACSGG | 40 cycles × (95 ℃, 30 s; 55 ℃, 30 s; 72 ℃,45 s) |  |
|  | R3cdR | GASTTCGGRTGSGTCTTGA |  |  |
| *nirK* | nirKF1aCuF | ATCATGGTSCTGCCGCG | 40 cycles × (95 ℃, 30 s; 53 ℃, 30 s; 72 ℃,45 s) |  |
|  | nirKR3CuR | GCCTCGATCAGRTTGTGGTT |  |  |
| *nosZ* | nosZ-1126F | GGGCTBGGGCCRTTGCA | 40 cycles × (95 ℃, 30 s; 53 ℃, 30 s; 72 ℃,45 s) |  |
|  | nosZ-1381R | GAAGCGRTCCTTSGARAACTTG |  |  |
| *Ustilaginoidea virens* | US3-3 | GCTCCAAGTGCGAGGATAACTGAAT | 35 cycles × (95 ℃, 30 s; 56 ℃, 30 s; 72 ℃,45 s) | Sun et al., 2013 |
|  | US1-5 | CCGGAGGATACAACCAAAAAAACTCT |  |  |
| *Magnaporthe oryza* | 28SMF | ACCCTACTGATGACCTCG | 35 cycles × (95 ℃, 30 s; 56 ℃, 30 s; 72 ℃,45 s) | Li et al., 2011 |
|  | 28SMR | GTGTCAAAATTACAATACGC |  |  |

**Supplementary Table 3.** The effects of straw retention on C and N-cycling enzyme activities

| **Treatment** | **C-Cycle** | | | **N-Cycle** | | |
| --- | --- | --- | --- | --- | --- | --- |
|  | **β-glucosidase (BG) (nmol h-1 g-1)** | **β-cellobiosidase (CBH)  (nmol h-1 g-1)** | **β-xylosidase (nmol h-1 g-1)** | **N-acetyl-glucosaminidase(NAG) (nmol h-1 g-1)** | **L-Leucine aminopeptidase (LAP) (μmol d-1 g-1)** | **urease  (μg d-1 g-1)** |
| SCK | 14.23±0.67a | 1.50±0.26a | 4.28 ±0.49c | 1.64±0.10bc | 70.36±2.08a | 384.22±10.92b |
| S1 | 15.39 ±0.52a | 1.87 ±0.25a | 4.78 ±0.27bc | 1.47 ±0.02c | 73.43±1.80a | 375.68±11.18b |
| S3 | 15.28±0.88a | 2.41±0.38a | 7.25±0.89a | 1.89±0.11b | 80.35±4.85a | 451.12±11.12a |
| S5 | 14.56±0.83a | 2.13±0.29a | 6.72±0.64ab | 2.38±0.08a | 71.52±3.43a | 438.29±5.31a |
| Note: Data are presented as x±s( n=3). Values at the same column followed by different lower case letters differed significantly at *P* < 0.05 (one-way analysis of variance). SCK, the straw was not returned to the field; S1, S3, and S5, the straw was returned to the field consecutively for 1, 3, and 5 years, respectively. | | | | | | |

**Supplementary Table 4.** The effects of straw retention on the copy numbers of the 16S rRNA and ITS genes, C and N cycling genes, and rice pathogen

| **Treatment** | **16S rRNA** （×109 copies/g） | **ITS1  (×10**8 copies/g) | ***cbh I* (×10**6 **copies/g)** | **GH48 (×10**7 copies/g) | ***sdimo* (×10**7 **copies/g)** | ***nifH* (×10**9 **copies/g)** | ***amoA*** (×107 copies/g) | ***amoB*** (×106 copies/g) | ***narG*** (×107 copies/g) | ***nirS*** (×108 copies/g) | ***nirK*** (×108 copies/g) | ***nosZ*** (×107 copies/g) | ***Ustilaginoidea virens*** （×104 copies） | ***Magnaporthe oryza***（×104 copies） |
| --- | --- | --- | --- | --- | --- | --- | --- | --- | --- | --- | --- | --- | --- | --- |
| SCK | 3.68±0.19b | 0.73±0.11c | 8.45±0.63a | 6.62±0.65a | 2.83±0.12c | 1.02±0.10b | 2.33±0.06c | 3.99±0.12b | 5.37±0.70a | 3.59±0.11a | 1.32±0.16a | 2.83±0.37a | 0.93±0.15b | 2.96±0.48c |
| S1 | 5.00±0.11a | 0.84±0.06bc | 7.62±0.55a | 5.47±0.32a | 3.50±0.28bc | 1.00±0.02b | 2.74±0.08c | 2.10±0.27b | 5.11±2.19a | 2.80±0.64ab | 1.06±0.45a | 2.21±0.89a | 1.15±0.09b | 5.64±1.17bc |
| S3 | 3.02±0.11b | 1.17±0.15a | 9.70±0.45a | 7.20±0.70a | 4.40±0.31a | 1.35±0.18ab | 7.13±0.23a | 6.03±0.50a | 7.99±2.54a | 3.25±0.39a | 1.11±0.43a | 3.31±0.77a | 2.36±0.57a | 10.70±0.90b |
| S5 | 3.74±0.52b | 1.01±0.23ab | 9.28±1.63a | 6.23±0.28a | 3.97±0.26ab | 1.49±0.16a | 3.85±0.32b | 5.21±0.09a | 6.28±0.68a | 2.12±0.08b | 0.97±0.19a | 3.19±0.62a | 2.81±0.18a | 13.28±3.48a |
| Note: Data are presented as x±s( n=3). Values at the same column followed by different lower case letters differed significantly at P < 0.05 (one-way analysis of variance). SCK, the straw was not returned to the field; S1, S3, and S5, the straw was returned to the field consecutively for 1, 3, and 5 years, respectively. | | | | | | | | | | | | | | |

**Supplementary Table 5.** Comparison of operational taxonomic units (OTUs) across different soil samples

|  | **Sample** | **Number of valid sequences** | **Number of different taxonomic categories** | | | | | |
| --- | --- | --- | --- | --- | --- | --- | --- | --- |
|  |  |  | **Phylum** | **Class** | **Order** | **Family** | **Genus** | **OTUs** |
| Bacteria | SCK_1 | 45616 | 48 | 154 | 330 | 471 | 740 | 4054 |
|  | SCK_2 | 47584 | 55 | 162 | 344 | 502 | 785 | 4228 |
|  | SCK_3 | 48422 | 51 | 159 | 339 | 484 | 797 | 4367 |
|  | S1_1 | 46449 | 47 | 151 | 324 | 453 | 713 | 3601 |
|  | S1_2 | 49604 | 47 | 156 | 315 | 441 | 696 | 3665 |
|  | S1_3 | 47848 | 50 | 155 | 325 | 469 | 740 | 3909 |
|  | S3_1 | 49288 | 46 | 150 | 332 | 483 | 815 | 4629 |
|  | S3_2 | 51410 | 47 | 146 | 308 | 448 | 736 | 4002 |
|  | S3_3 | 48119 | 51 | 151 | 327 | 470 | 775 | 3958 |
|  | S5_1 | 47661 | 53 | 159 | 336 | 475 | 765 | 4050 |
|  | S5_2 | 45666 | 50 | 148 | 320 | 472 | 784 | 4135 |
|  | S5_3 | 47213 | 51 | 154 | 327 | 475 | 767 | 3906 |
|  | SCK_1 | 58333 | 9 | 28 | 47 | 84 | 110 | 510 |
| Fungi | SCK_2 | 54323 | 10 | 26 | 45 | 81 | 104 | 363 |
|  | SCK_3 | 59785 | 14 | 32 | 58 | 96 | 120 | 593 |
|  | S1_1 | 61268 | 10 | 28 | 49 | 83 | 106 | 374 |
|  | S1_2 | 49869 | 9 | 25 | 44 | 76 | 92 | 394 |
|  | S1_3 | 68823 | 11 | 30 | 56 | 92 | 120 | 481 |
|  | S3_1 | 82369 | 12 | 32 | 60 | 115 | 174 | 561 |
|  | S3_2 | 65834 | 7 | 25 | 46 | 86 | 118 | 333 |
|  | S3_3 | 67766 | 9 | 28 | 54 | 99 | 152 | 481 |
|  | S5_1 | 69526 | 9 | 30 | 53 | 93 | 123 | 465 |
|  | S5_2 | 72351 | 10 | 31 | 55 | 94 | 123 | 510 |
|  | S5_3 | 72336 | 9 | 27 | 49 | 79 | 97 | 357 |
| Note: SCK, the straw was not returned to the field; S1, S3, and S5, the straw was returned to the field consecutively for 1, 3, and 5 years, respectively. | | | | | | | | |

**Supplementary Table 6.** Relative abundance of dominant soil taxa at the phylum level within each bacterial-fungal meta-network

| **Phyla** | | **Treatment** | | | |
| --- | --- | --- | --- | --- | --- |
|  |  | **SCK** | **S1** | **S3** | **S5** |
| Bacteria | Chloroflexi | 27.55±1.12a | 24.81±2.72a | 28.67±3.16a | 22.80±2.42a |
|  | Proteobacteria | 13.72±0.62b | 14.60±1.00ab | 13.94±1.40b | 18.39±1.66a |
|  | Actinobacteriota | 12.24±0.70b | 15.74±0.82a | 15.74±1.05a | 15.84±0.30a |
|  | Acidobacteriota | 14.09±1.52a | 13.98±1.16a | 15.66±2.04a | 10.48±1.53a |
|  | Bacteroidota | 4.65±0.69ab | 3.93±0.43ab | 3.55±0.36b | 5.68±0.64a |
|  | Desulfobacterota | 4.41±0.36ab | 4.15±0.27ab | 3.29±0.19b | 5.20±0.86a |
|  | MBNT15 | 4.37±0.44a | 4.07±0.62ab | 2.43±0.56b | 3.06±0.43ab |
|  | Myxococcota | 2.52±0.18b | 2.63±0.36b | 2.81±0.06ab | 3.36±0.13a |
|  | Nitrospirota | 2.94±0.18ab | 3.52±0.54a | 1.80±0.14b | 2.06±0.44b |
|  | Firmicutes | 1.75±0.26b | 2.46±0.23ab | 2.04±0.16b | 3.40±0.68a |
|  | Gemmatimonadota | 2.32±0.15a | 2.54±0.37a | 2.18±0.36a | 2.01±0.22a |
|  | Patescibacteria | 1.71±0.08a | 1.52±0.17a | 1.27±0.13a | 1.65±0.14a |
|  | Verrucomicrobiota | 0.83±0.15a | 0.80±0.16a | 1.00±0.11a | 0.59±0.12a |
|  | Latescibacterota | 1.01±0.09a | 0.68±0.02ab | 0.83±0.17ab | 0.54±0.14b |
|  | Methylomirabilota | 0.57±0.01ab | 0.53±0.03b | 0.79±0.14a | 0.44±0.07b |
|  | Others | 5.30±0.21a | 4.06±0.18b | 3.99±0.21b | 3.79±0.17b |
| Fungi | Ascomycota | 29.44±4.29c | 34.45±6.02bc | 46.95±3.99ab | 57.42±2.32a |
|  | Basidiomycota | 26.32±4.43a | 31.52±6.40a | 29.90±6.29a | 20.82±4.65a |
|  | Mortierellomycota | 19.49±7.98a | 19.98±9.12a | 9.85±1.17a | 11.09±2.58a |
|  | unclassified_k_Fungi | 14.51±2.81a | 9.20±2.37a | 9.02±3.45a | 6.43±1.11a |
|  | Chytridiomycota | 5.15±1.00a | 3.57±0.68a | 2.81±0.75a | 2.91±0.51a |
|  | Monoblepharomycota | 1.14±0.23a | 0.61±0.05b | 0.41±0.12b | 0.40±0.11b |
|  | Blastocladiomycota | 2.46±2.40a | 0.02±0.01a | 0.02±0.02a | 0±0.00a |
|  | Rozellomycota | 0.51±0.33a | 0.10±0.05a | 0.69±0.35a | 0.26±0.19a |
|  | Others | 0.98±0.09a | 0.56±0.05b | 0.34±0.13b | 0.67±0.12ab |
| Note: Data are presented as x±s( n=3). Values at the same row followed by different lower case letters differed significantly at *P* < 0.05 (one-way analysis of variance). SCK, the straw was not returned to the field; S1, S3, and S5, the straw was returned to the field consecutively for 1, 3, and 5 years, respectively. | | | | | |

**Supplementary Table 7.** Relative abundance of dominant soil taxa at the genera level within each bacterial-fungal meta-network

| **Genus** | | **Treatment** | | | |
| --- | --- | --- | --- | --- | --- |
|  |  | **SCK** | **S1** | **S3** | **S5** |
| Bacteria | *norank_f__Anaerolineaceae* | 4.34±0.74a | 4.35±1.14a | 5.14±1.32a | 3.69±0.78a |
|  | *norank_f__norank_o__Vicinamibacterales* | 3.82±0.66a | 3.89±0.75a | 5.08±0.84a | 2.56±0.65a |
|  | *Pseudarthrobacter* | 1.76±0.16a | 1.87±0.18a | 2.04±0.22a | 1.89±0.38a |
|  | *norank_f__norank_o__norank_c__Thermodesulfovibrionia* | 1.99±0.07ab | 2.36±0.41a | 1.00±0.13b | 2.00±0.41ab |
|  | *unclassified_f__Intrasporangiaceae* | 1.56±0.16a | 1.64±0.20a | 1.83±0.44a | 1.88±0.04a |
|  | *norank_f__Bacteroidetes_vadinHA17* | 1.21±0.16a | 1.54±0.16a | 1.74±0.07a | 1.91±0.55a |
|  | *Marmoricola* | 0.73±0.10a | 1.63±0.55a | 1.06±0.14a | 1.36±0.47a |
|  | *norank_f__Vicinamibacteraceae* | 1.13±0.27a | 0.70±0.17a | 0.75±0.68a | 0.66±0.25a |
|  | *Trichococcus* | 0.04±0.02b | 0.07±0.00b | 0.27±0.12b | 1.37±0.50a |
|  | *Bradyrhizobium* | 0.68±0.04bc | 0.82±0.09ab | 0.57±0.00c | 0.96±0.04a |
| Fungi | *Tausonia* | 13.29±2.75a | 5.13±1.77b | 13.94±2.07a | 11.21±3.05a |
|  | *Mortierella* | 7.89±2.34b | 17.65±2.94a | 4.80±0.47b | 6.71±1.37b |
|  | *Mrakia* | 5.28±1.32b | 16.74±4.01a | 4.66±0.94b | 4.56±0.73b |
|  | *Pseudeurotium* | 6.39±2.16b | 5.71±2.98b | 2.52±0.40b | 16.39±1.67a |
|  | *Psilocybe* | 4.78±0.71a | 2.02±0.25a | 8.54±5.87a | 1.45±0.38a |
|  | *Pseudogymnoascus* | 0.13±0.02b | 1.38±0.75b | 0.49±0.17b | 12.83±0.70a |
|  | *unclassified_o__Sordariales* | 0.76±0.01b | 5.58±1.62a | 4.52±0.38a | 4.85±1.27a |
|  | *Mortierellales_gen_Incertae_sedis* | 2.47±1.08a | 1.83±1.10a | 3.50±1.22a | 3.78±1.68a |
|  | *unclassified_p__Chytridiomycota* | 3.49±0.56a | 2.84±0.48a | 1.81±0.76a | 1.92±0.46a |
|  | *Trichosporiella* | 0.14±0.06b | 0.24±0.22b | 3.42±0.54a | 5.61±0.32a |
|  | *Achroiostachys* | 1.17±0.22ab | 0.45±0.19b | 1.88±0.66ab | 3.83±0.86a |
|  | *unclassified_f__Mortierellaceae* | 0.91±0.44a | 0.27±0.08a | 0.75±0.21a | 0.15±0.13a |
|  | *Cladosporium* | 1.35±0.67a | 1.93±0.77a | 2.15±1.34a | 0.54±0.17a |
|  | *unclassified_o__Pleosporales* | 1.18±0.72a | 1.28±0.43a | 2.82±0.99a | 0.55±0.23a |
|  | *unclassified_o__Trichosphaeriales* | 0.99±0.27a | 0.97±0.55a | 2.47±1.62a | 0.53±0.14a |
|  | *Stellatospora* | 3.01±1.25a | 1.16±0.41ab | 0.56±0.27b | 0.36±0.18b |
|  | *Acremonium* | 2.53±1.64a | 0.26±0.16a | 0.39±0.17a | 1.33±0.97a |
|  | *unclassified_o__Agaricales* | 0.13±0.05a | 2.83±1.61a | 0.16±0.07a | 1.03±0.29a |
|  | *Linnemannia* | 3.17±1.12a | 0.03±0.03b | 0.67±0.53b | 0.02±0.02b |
|  | *Clonostachys* | 0.21±0.09a | 0.78±0.73a | 2.02±0.98a | 0.84±0.66a |
|  | *Alternaria* | 0.28±0.14a | 2.48±1.89a | 0.93±0.55a | 0.15±0.04a |
|  | *Sarocladium* | 0.59±0.29a | 0.46±0.33a | 2.57±1.45a | 0.21±0.11a |
|  | *Peziza* | 0.02±0.02a | 0.01±0.00a | 0.54±0.20a | 1.20±0.78a |
|  | *Glaciozyma* | 0.48±0.14b | 1.99±0.67a | 0.35±0.12b | 0.51±0.10b |
|  | *Podospora* | 0.68±0.27a | 1.30±0.98a | 0.93±0.22a | 0.4±0.14a |
|  | *Solicoccozyma* | 0.47±0.15a | 1.37±0.95a | 0.66±0.16a | 0.12±0.06a |
|  | *Clavidisculum* | 0.20±0.05b | 0.01±0.00b | 0.72±0.37ab | 1.53±0.73a |
|  | *Schizothecium* | 0.20±0.03b | 0.04±0.02b | 1.37±0.57a | 0.74±0.43ab |
|  | *Ustilaginoidea* | 0.91±0.26a | 0.12±0.02a | 1.23±0.74a | 0.06±0.02a |
|  | *Fusarium* | 0.16±0.08a | 0.09±0.07a | 1.06±0.68a | 0.04±0.02a |
|  | *unclassified_c__Sordariomycetes* | 0.15±0.09a | 0.05±0.04a | 1.00±0.69a | 0.08±0.03a |
| Note: Data are presented as x±s( n=3). Values at the same row followed by different lower case letters differed significantly at *P* < 0.05 (one-way analysis of variance). SCK, the straw was not returned to the field; S1, S3, and S5, the straw was returned to the field consecutively for 1, 3, and 5 years, respectively. | | | | | |
